# C-SOM SNN: A Continuous Self-Organizing Map Spiking Neural Network Based on STDP Unsupervised Learning Encoding with Designed Lateral Inhibition Distribution

**DOI:** 10.3390/biomimetics11100727

**Published:** 2026-10-11

**Authors:** Lingfei Mo, Xin Liu, Mengting Tang

**Affiliations:** State Key Laboratory of Comprehensive PNT Network and Equipment Technology, School of Instrumentation Science and Engineering, Southeast University, Nanjing 210096, China; 230228955@seu.edu.cn (X.L.); 220233582@seu.edu.cn (M.T.)

**Keywords:** Spiking Neural Network (SNN), STDP, lateral inhibition distribution, Self-Organizing Map (SOM), unsupervised learning, encoding

## Abstract

To address traditional Spiking Neural Networks (SNNs) limitations in continuous variable encoding and biological plausibility, this study proposes C-SOM SNN, a novel SNN integrating SOM SNN clustering, CANN (Continuous Attractor Neural Network) continuous representation, and STDP unsupervised learning with good biological plausibility inspired by hippocampal spatial-coding neuron studies. Unlike conventional models confined to discrete variables, C-SOM SNN innovatively enables dual encoding of discrete and continuous variables via hybrid topology and Multi-Spike WTA (Winner-Take-All). Experiments validate its superiority on MNIST with fixed official train-test partition (five repeated runs with fixed seeds), and C-SOM SNN achieves 98.00 ± 0.18% accuracy versus 84.00 ± 0.40% (SOM-SNN) and 92.00 ± 0.31% (SNN-CANN). On Urban8K with official stratified 10-fold splits, it reaches 87.00 ± 0.65%, compared with 78.00 ± 0.92% (SOM-SNN) and 83.00 ± 0.84% (SNN-CANN), yielding measurable performance gains over baselines. In localization navigation, it stably encodes continuous landmark positions. It shows certain disjoint-cross-modal processing capability for visual and audio inputs in separate tasks by handling raw visual samples and audio signals transformed into image-like feature matrices. This work advances brain-inspired computing, offering an efficient, interpretable framework for complex tasks, with great potential for real-world disjoint-multi-modal applications.

## 1. Introduction

The neural basis of spatial cognition relies on a sophisticated network of specialized neurons in the hippocampal formation and surrounding regions. O’Keefe’s seminal discovery of place cells in 1971 revealed that hippocampal neurons fire selectively at specific locations, forming the foundation of cognitive maps through population coding mechanisms (O’Keefe & Dostrovsky, 1971 [1]). Complementing this finding, Moser and colleagues identified grid cells in the medial entorhinal cortex (MEC) in 2005, which generate hexagonal firing patterns to support metric spatial representation and path integration (Hafting et al. 2005 [2]). These discoveries established the core components of the brain’s “internal GPS system”, for which they were awarded the 2014 Nobel Prize. Theoretical frameworks have sought to explain these spatial coding mechanisms, with Lateral Inhibition Neural Networks providing a prominent model for grid cell activity. Samsonovich and McNaughton’s 1997 model demonstrated how recurrent synaptic connections could maintain stable spatial representations through path integration, accounting for various properties of place cell activity (Samsonovich & McNaughton, 1997 [3]). Recent advancements in cognitive space transformation models further elucidated interactions between place cells and grid cells in encoding local versus global spatial frames (Zhang et al. 2024 [4]). This spatial coding system is complemented by additional cell types: head direction cells in MEC layers II/III provide directional reference frames through multimodal integration of vestibular and visual cues (Giocomo et al. 2014 [5]), while boundary cells encode environmental edges to constrain spatial representations. A 2024 study identified novel boundary cells in the medial prefrontal cortex lacking theta rhythmicity, expanding our understanding of extra-hippocampal contributions to spatial cognition (Long et al. 2024 [6]). Recent modeling work further demonstrates that continuous attractor dynamics within MEC microcircuits underpin hexagonal grid cell firing patterns and stable spatial path integration (Chen et al. 2026 [7]). Another 2026 investigation reveals that grid cell signals mitigate spatial aliasing and complement boundary cell inputs to improve the reliability of hippocampal place representations (Johnson et al. 2026 [8]). Complementarily, new computational evidence shows that hippocampal place cell reorganization arises from integrated multi-source entorhinal inputs, supporting flexible spatial coding (Kuniyoshi et al. 2026 [9]). Recent advances in biomimetic computational intelligence effectively translate natural biological behaviors into feasible computational paradigms (Ghiaskar et al. 2024 [10]). Standardized evaluation protocols are also essential to ensure credible performance assessment of diverse biomimetic algorithms (Ghiaskar & Taheri, 2026 [11]). Furthermore, biomimetic neural investigations validate that tuned inhibitory interactions enable robust continuous variable coding (Hughes et al. 2026 [12]). Under this research context, this work proposes a ring-structured SNN with customized lateral inhibition to realize unified discrete and continuous variable encoding.

In computational neuroscience, Spiking Neural Networks (SNNs) have emerged as promising tools for modeling these biological mechanisms. Leveraging Spike-Timing-Dependent Plasticity (STDP), SNNs can implement unsupervised learning of spatial features with high energy efficiency (Safa et al. 2024 [13]). However, conventional models struggle to replicate the dynamic reorganization of spatial codes observed in vivo. Recent innovations, such as combining Self-Organizing Map (SOM) clustering with STDP, have shown potential for improving topological preservation of spatial representations (Fois et al. 2023 [14]). Neuromorphic implementations of these models on specialized hardware have demonstrated significant advantages, with energy efficiency improvements of 2–3 orders of magnitude compared to traditional artificial neural networks (Chen et al. 2025 [15]). Despite these advances, bridging biological realism and computational efficiency remains challenging, particularly in modeling the flexible remapping of spatial codes across environments (Latuske et al. 2018 [16]).

Traditional visual SLAM navigation systems face notable drawbacks in dynamic environments. DyPho-SLAM research shows substantial pose estimation errors when dynamic objects dominate the field of view (Liu et al. 2025 [17]), and industrial AGV navigation studies report severe feature loss under extreme lighting (Botomba et al. 2026 [18]). Lightweight SLAM variants such as COSMO still struggle to balance accuracy and speed on low-power hardware (Li et al. 2026 [19]). Fundamentally, heavy geometric computation brings high energy overhead (Cao et al. 2023 [20]), and the reliance on preconstructed maps limits adaptation to scene changes (Hroob et al. 2025 [21]).

**Figure 1:**
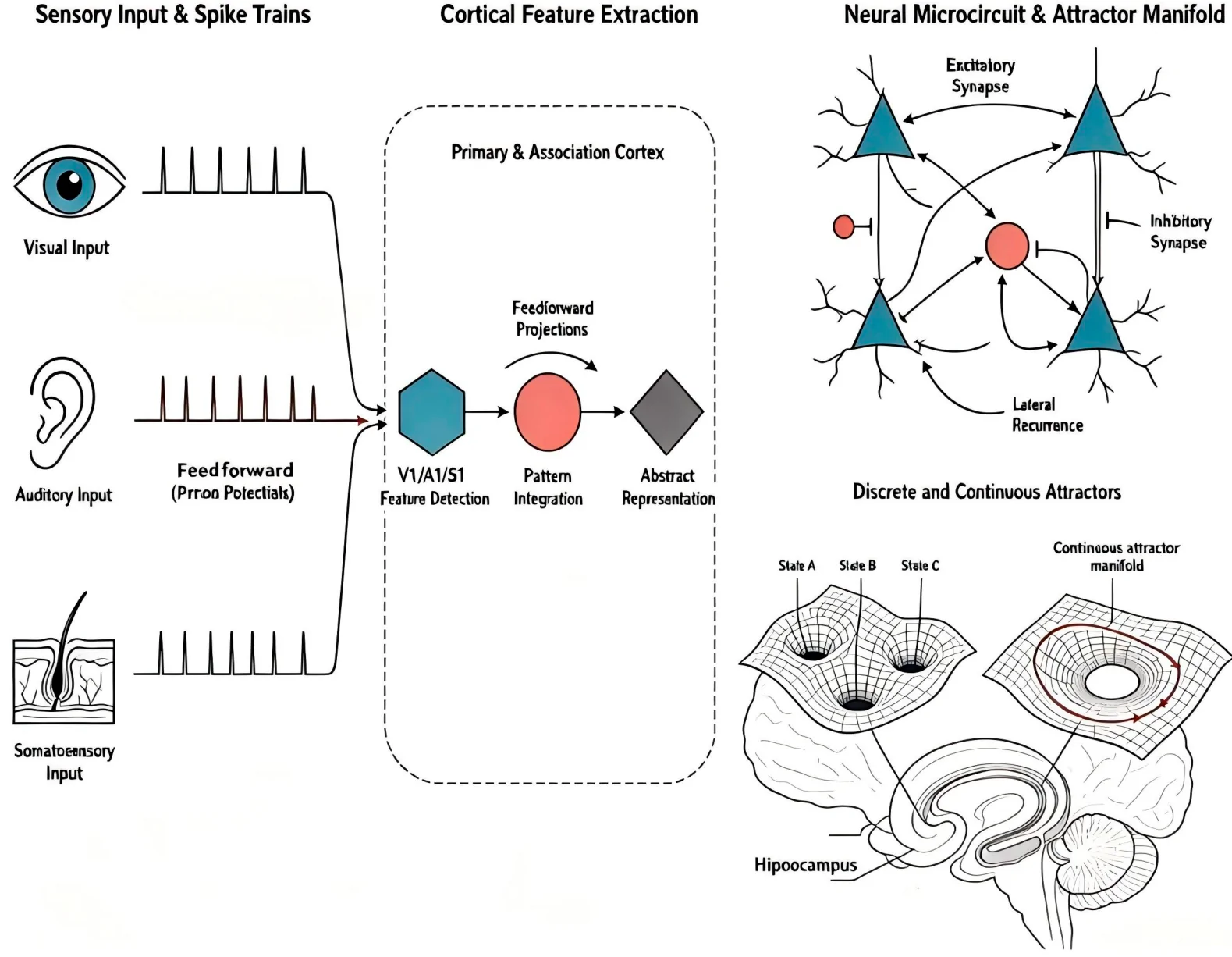
Schematic diagram of the brain’s spatial cognition system and C-SOM SNN architecture. The neural circuit diagram of brain spatial cognition is adapted from Bernacchia (Bernacchia, 2007 [23]).

As shown in Figure 1, brain-inspired navigation coding sidesteps these pitfalls by borrowing a few tricks from neuroscience, delivering three headline benefits in one package. Energy budgets shrink dramatically when Spiking Neural Networks take the wheel: on Intel’s Loihi chip, a neuromorphic agent zig-zagging without a map burns 75% less juice per inference than a GPU-powered cousin, thanks to the frugal gossip of spike-timing codes (Hao et al. 2026 [22]). A Hebbian plastic network learns discrete or continuous attractors based on stimulus statistics, and mismatched sensory inputs destabilize continuous attractors into discrete ones (Bernacchia, 2007 [23]). Best of all, the rigidity of pre-built maps disappears: by fusing whatever the sensors whisper—visual, vestibular, proprioceptive—these models learn the world on the fly. Queensland University of Technology’s neuromorphic rig recently wandered through never-seen-before rooms with no map whatsoever, self-assembling a coherent spatial memory via plain unsupervised learning (Bai et al. 2024 [24]), a neat counter-example to semantic SLAM systems that keep merging look-alike chairs and corrupting their own maps (Bian et al. 2025 [25]). The pivot is simple—swap geometric feature counting for the brain’s own cognitive-map playlist—and suddenly robust navigation arrives with a sliver of the usual energy bill (Huang et al. 2025 [26]).

This work proposes a novel C-SOM SNN framework that enhances variable encoding performance via unsupervised learning and neuromorphic dynamics. The core innovations are summarized as follows: (1) Unlike conventional spiking networks with fixed inhibition patterns, C-SOM SNN integrates STDP unsupervised learning with spatially customized lateral inhibition. The distance-enhanced inhibition mechanism is embedded into STDP weight updates, enabling stable extraction of spatiotemporal feature correlations following input sparse coding. (2) A hybrid topological encoding paradigm is established by combining SOM competitive dynamics with tailored lateral inhibition. Local weak inhibition facilitates synchronous spiking of adjacent neurons for similar inputs, while long-range strong inhibition and SOM competition constrain weight optimization to winning neurons and their neighbors. This produces spatially continuous, stable spike clusters with reliable topological preservation and noise robustness. (3) A unified dual-mode encoding scheme is implemented under a single learnable network structure. Strong inhibition generates independent spike clusters for discrete classification via orthogonal sparse coding, whereas moderate inhibition enables adjacent cluster co-activation to form continuous spike manifolds for continuous variable encoding. This design eliminates the inherent trade-off between discrete classification and continuous spatial mapping in existing SNNs, allowing adaptive generation of isolated or continuous spike topographies for diverse inputs.

The remainder of this paper is organized as follows: Section 2 reviews related literature and performs comparative summarization. Representative models including Diehl-SNN, traditional SOM, and SNN-CANN are summarized and compared across multiple dimensions, clarifying research limitations and motivating the design of the proposed model. Section 3 details the full methodological pipeline of the proposed C-SOM SNN, including the adaptive LIF neuron model, customized lateral inhibition distribution rule, event-driven sparse coding strategy, SOM clustering algorithm, and overall ring network structure. Section 4 presents comprehensive experiments on discrete audio classification and continuous spatial localization tasks to validate model performance. Section 5 further discusses the internal mechanism, comparative superiority, hyperparameter influence and existing limitations of the model. Section 6 concludes the overall work and illustrates future research directions.

## 2. Related Work

This subsection reviews brain-inspired spatial coding and unsupervised learning studies to identify research gaps motivating C-SOM SNN. Diehl’s lateral inhibition Diehl-SNN implements spike-based competitive feature extraction but requires manually tuned inhibition without adaptive weight learning. Traditional SOM produces topological clustering of high-dimensional features yet operates on rate-coded signals and lacks spike-timing plasticity. SNN-CANN supports continuous representation and hardware-oriented simplification but still relies on manual parameter configuration. Collectively, these works demonstrate the promise of neuromorphic designs while revealing the need for a spiking network integrating inhibition competition, SOM-style topological clustering and biologically plausible adaptive synaptic updates. To systematically illustrate the distinctions among these brain-inspired models, this study conducts comparisons across four key dimensions: coding paradigm, learning mechanism, network architecture, and topological/continuous representation capability. (1) Coding paradigm: Diehl-SNN adopts spike-based discrete feature coding, traditional SOM relies on rate-coded static features, and SNN-CANN supports spike-based continuous attractor coding; by contrast, C-SOM SNN implements a unified spike-based discrete-continuous coding scheme. (2) Learning mechanism: Diehl-SNN uses fixed lateral inhibition without adaptive synaptic updates, traditional SOM achieves unsupervised clustering but lacks spike-timing-dependent plasticity, and SNN-CANN builds on fixed recurrent attractor dynamics; our C-SOM SNN leverages STDP for adaptive weight learning combined with SOM neighborhood competition. (3) Network architecture: Diehl-SNN is a feedforward spiking network with hand-tuned inhibition, traditional SOM has grid topology without recurrent spiking connections, and SNN-CANN depends on predefined attractor weight matrices; C-SOM SNN adopts a ring-structured output layer equipped with explicit recurrent connections and configurable lateral inhibition. (4) Parameter adaptability and representation: Diehl-SNN, traditional SOM and SNN-CANN all require heavy manual parameter tuning and cannot simultaneously realize topological clustering and continuous spatial encoding, while the proposed C-SOM SNN reduces manual tuning and integrates SOM-like topological clustering with CANN-style continuous representation.

### 2.1. Diehl Lateral Inhibition STDP SNN

**Figure 2:**
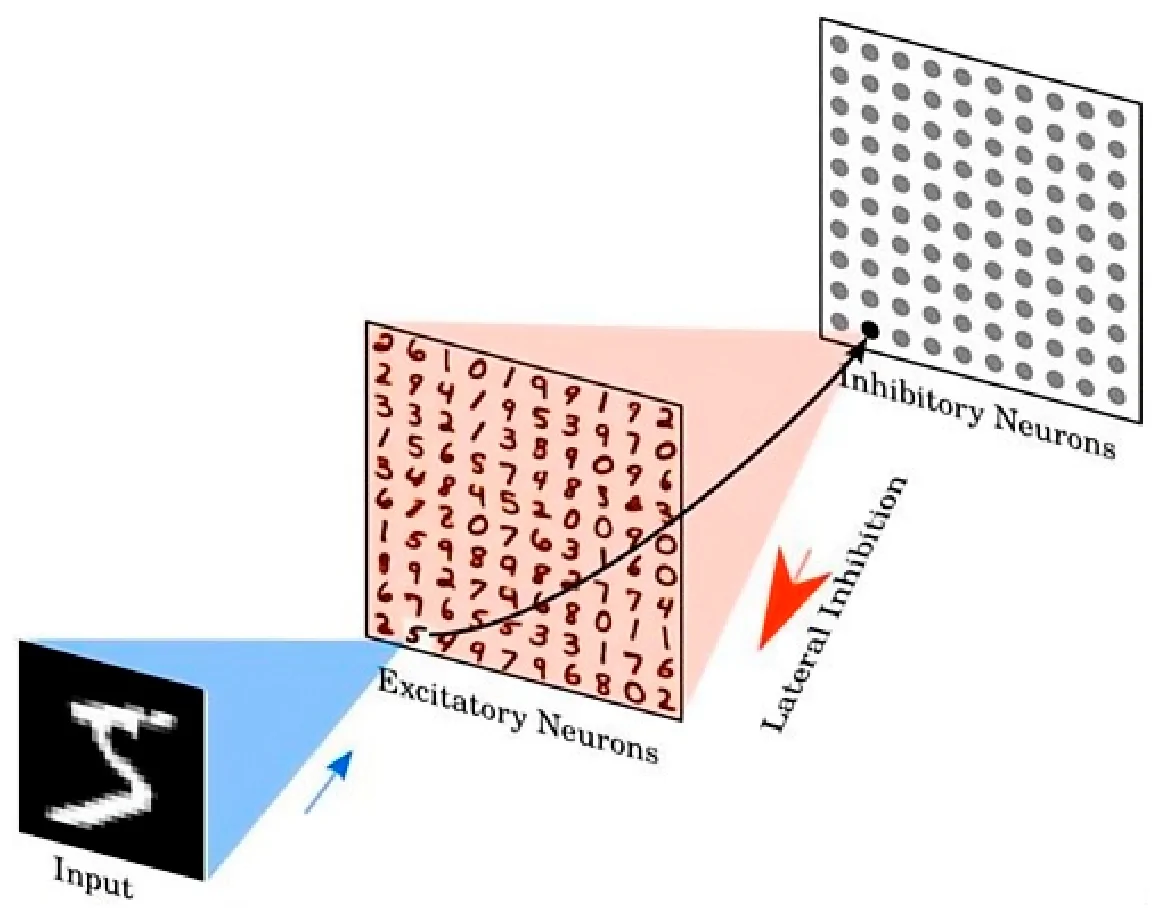
Architecture diagram of Diehl unsupervised STDP SNN with lateral inhibition (Diehl & Cook, 2015 [27]). The blue arrow denotes the input direction.

Diehl and Cook’s unsupervised STDP Diehl-SNN with classic lateral inhibition (Diehl & Cook, 2015 [27]) is a pioneering model for digit recognition, whose architecture (as illustrated in Figure 2) integrates spike-timing-dependent plasticity (STDP) and lateral inhibition to realize unsupervised feature learning. The system takes visual inputs (e.g., MNIST digits) and transmits them to a layer of excitatory neurons, which interact with a layer of inhibitory neurons via lateral inhibition connections. In this framework, lateral inhibition operates through a competitive mechanism: activated excitatory neurons send signals to inhibitory neurons, which suppress the activity of other excitatory neurons. This competition ensures that only a sparse subset of excitatory neurons fire for each input, facilitating efficient and discriminative feature coding. The STDP rule adjusts the weights between input and excitatory neurons based on spike timing, enabling the network to learn digit representations in an unsupervised manner. The core value of this architecture resides in how lateral inhibition drives sparse, orthogonal coding—each digit evokes a distinct pattern of excitatory neuron activity, allowing the network to achieve effective digit recognition. This work showcases the efficacy of combining STDP and lateral inhibition for unsupervised learning, yet it mainly focuses on discrete variable (digit) classification and lacks mechanisms for continuous variable encoding. These limitations motivate our research to develop advanced SNN architectures that extend such principles to handle both discrete and continuous variables.

### 2.2. SOM Unsupervised Learning Algorithm

**Figure 3:**
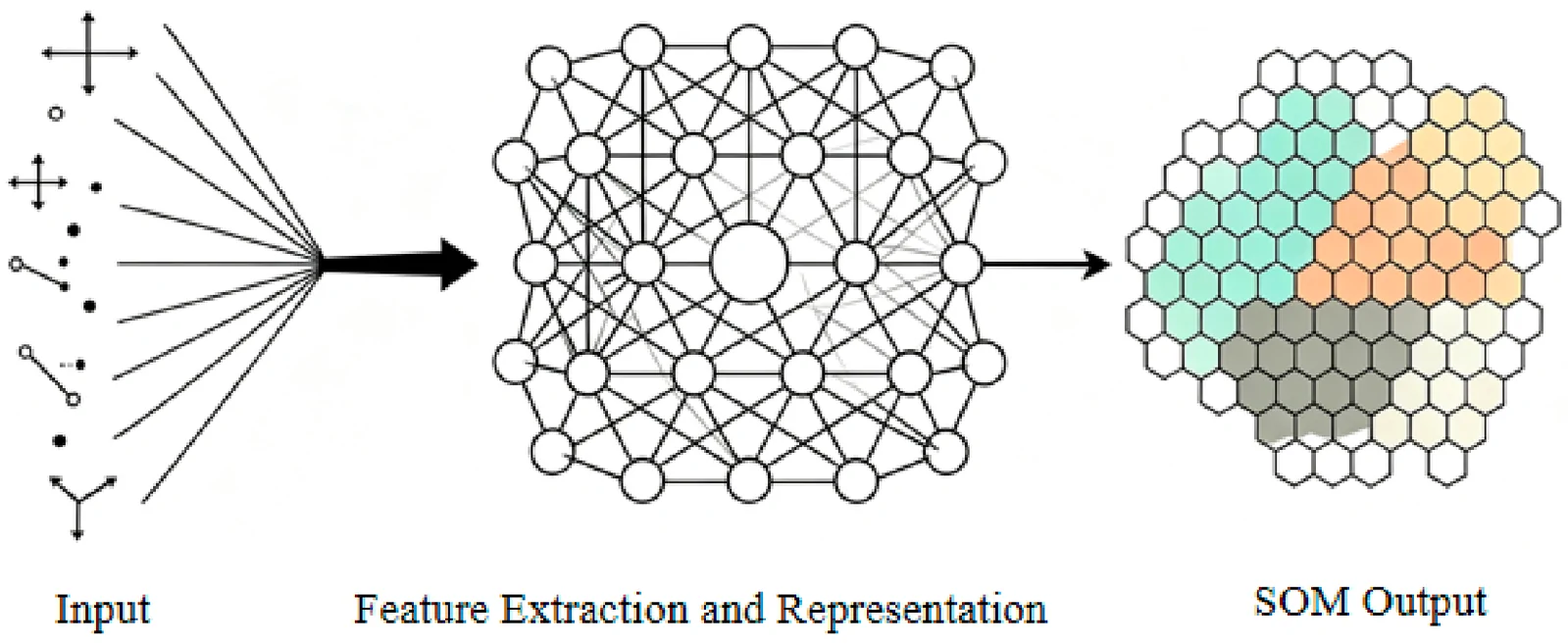
Schematic diagram of the SOM unsupervised learning algorithm (Kohonen, 2013 [28]). Shapes in the input layer indicate various inputs, and colors in the output layer correspond to different clusters.

As shown in Figure 3, the mapping of four document classes (Corporate-industrial, Economics and economic indicators, Government and social, Securities and commodities trading) onto the 2000 node SOM (Kohonen, 2013 [28]) presents a clear topological order. Hit densities (matches between documents and SOM models) are visualized via gray shades, with each class concentrated in coherent regions, and Classes 1, 3, and 4 even forming single-connected clusters despite their closely related topics. The SOM unsupervised learning algorithm can project high-dimensional data into a regular two-dimensional vector grid to implement data clustering and similarity analysis. During training, each input sample matches the optimal winning neuron, and synaptic vectors of both the winning neuron and its neighboring units are updated synchronously. Such neighborhood adaptive optimization enables similar feature vectors to aggregate spatially and dissimilar features to be distanced, forming structured topological feature distribution. Different from conventional clustering methods such as k-means and vector quantization that merely partition data without spatial topological constraints, SOM preserves inherent inter-sample similarity and continuous feature transitions. Therefore, SOM effectively compensates for the poor visualization and structural representation defects of traditional unsupervised clustering approaches for high-dimensional data.

### 2.3. SNN-CANN

Continuous Attractor Neural Networks (CANNs) have emerged as a canonical model for neural information representation, particularly in encoding continuous variables such as spatial position, head direction, and visual orientation (Wu et al. 2016 [29]). At the core of CANN’s functionality is its recurrent connectivity architecture, where local excitatory connections between neurons are balanced by global inhibitory mechanisms to form a stable dynamic system. As illustrated in the accompanying diagram, this network topology generates a continuous manifold of attractor states, manifested as Gaussian-shaped activity bumps that maintain stable firing patterns through mutual feedback loops. These activity bumps can shift smoothly over the neuron population under external inputs, enabling high-precision encoding of continuous physical quantities.

**Figure 4:**
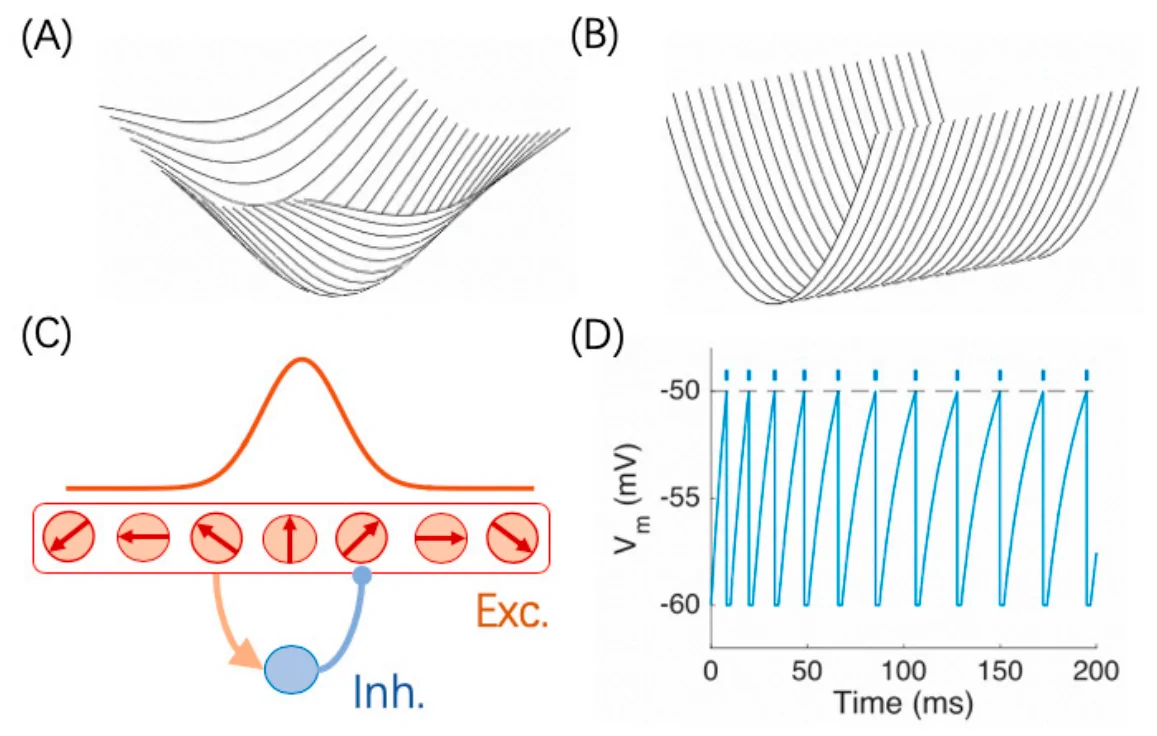
(**A**) A discrete attractor; (**B**) a bunch of continuous attractors; (**C**) the structure of a SNN-CANN (Spiking Continuous Attractor Neural Network) integrated with Spike Frequency Adaptation (SFA), the arrows denote the preferred direction of each neuron, and the orange curve represents the activity bump profile; (**D**) example of a single excitatory neuron’s response in the SNN-CANN with SFA (Yu et al. 2019 [30]).

As shown in Figure 4, SNN-CANN (Yu et al. 2019 [30]) advances the fusion of spiking neural network (SNN) dynamics and continuous attractor mechanisms, yet its application to anticipative tracking remains underexplored due to the inherent complexity of spiking neurons and conductance-based synaptic currents—even though the theoretical framework linking continuous attractor dynamics to spatial and temporal encoding is well-established, the computational overhead of biophysically detailed spiking models hinders translation into functional anticipative systems. Traditional SNN-CANN architectures, which rely on intricate recurrent connections and dense synaptic matrices (as illustrated in related diagrams), also complicate neuromorphic hardware implementation and limit real-time performance; while recent efforts have simplified SNN-CANNs for neuromorphic deployment to achieve path integration, these streamlined models lack anticipative tracking functionality, a critical gap for dynamic navigation tasks. To address this, the study developed a novel SNN-CANN integrated with Spike Frequency Adaptation (SFA)—a biological mechanism where neuron firing rates adjust to sustained inputs to enable predictive responses to moving stimuli—with the SFA module embedded in the network’s recurrent layer to modify spiking neuron threshold dynamics, generating forward-shifted activity bumps for anticipative tracking.

## 3. Methodology and Theoretical Derivation

This section details the methodological framework integrating SOM with a designed lateral inhibition distribution rule for neuromorphic learning. The core is this rule: distance-dependent inhibition (weak for adjacency, strong for distance) ensures SOM-clustered neuron groups of the same category stay spatially adjacent, laying the foundation for continuous encoding. It integrates STDP unsupervised learning and event-driven sparse coding, and designs a hierarchical structure combined with multi-spike WTA, supporting both discrete classification and continuous representation to underpin multimodal tasks.

### 3.1. Adaptive LIF Neuron Model with Lateral Inhibition

**Figure 5:**
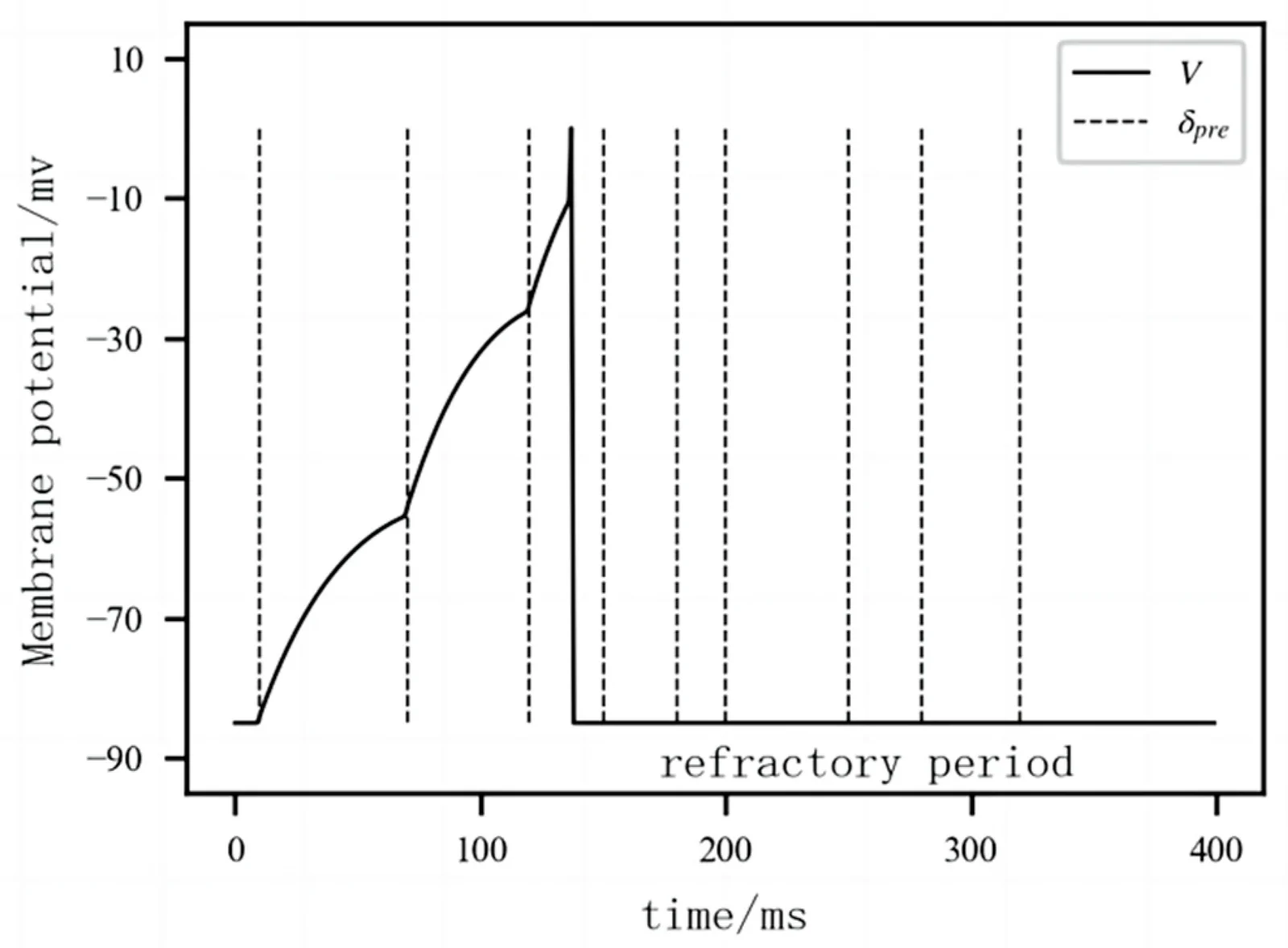
Membrane potential dynamics of an adaptive LIF neuron with dynamic threshold, showing depolarization, spiking, and the ensuing refractory period.

This section introduces the adaptive Leaky Integrate-and-Fire (LIF) neuron model with lateral inhibition, which emulates biological neurons’ dynamic threshold adjustment and competitive interaction mechanisms to support spatial information processing. As shown in Figure 5, the membrane potential of the adaptive LIF neuron (solid line) exhibits dynamics of integrating synaptic inputs while undergoing leakage—a slow decay in potential when no input is received. Initially, the membrane potential rests at a negative value (around −85 mV). Upon receiving excitatory inputs, it depolarizes (rises) over time: from 0 to about 120 ms, the potential gradually climbs from −85 mV toward the firing threshold. When the membrane potential reaches a dynamic threshold (modulated by signals like delta_pre, shown as the dashed line and reflecting adaptive changes), the neuron generates an action potential (spike). Immediately after spiking, the neuron enters a refractory period (indicated by the flat segment of the membrane potential after the spike, lasting until about 150 ms), during which the membrane potential resets to a low value and becomes insensitive to inputs—this mimics biological neurons’ short-term inhibition to prevent excessive firing. The adaptive property is embodied in the dynamic threshold: unlike static-threshold LIF models, this threshold evolves with the neuron’s activity history. For example, frequent spiking elevates the threshold (increasing the difficulty of subsequent firing, simulating neuronal fatigue), while sparse activity lowers the threshold (enhancing sensitivity). Meanwhile, lateral inhibition is implemented via inhibitory synaptic connections between neurons: when a neuron depolarizes or spikes, it sends inhibitory signals to neighboring neurons, suppressing their membrane potential elevation. This “near-excitation, far-inhibition” mechanism sharpens the contrast of neural activity—analogous to lateral inhibition in the visual system that enhances edge perception—ensuring only the most strongly activated neuron (or a sparse set of neurons) fires, while others are inhibited.

The adaptive LIF (ALIF) neuron model with lateral inhibition encapsulates three key biological features—membrane potential integration, dynamic threshold modulation, and inter-neuronal competitive inhibition—through a set of interlinked equations. The first equation governs the core dynamics of membrane potential evolution when the neuron has not yet reached its firing threshold, capturing the interplay between synaptic inputs, intrinsic membrane properties, and regulatory currents that shape the neuron activation state. Equation (1) formalizes this relationship as follows (this formula is adopted from prior research (You et al. 2022 [31])):

(1) $$\frac{dV}{dt}=\frac{1}{C_{m}}\cdot \left[-g_{L}\cdot (V-V_{L})+I_{exc}-I_{inh\backslash \_lat}-I_{adap}\right]$$

In this equation, the left-hand term *dV*/*dt* represents the rate of change of the membrane potential (*V*) over time, dictating whether the neuron undergoes depolarization (a rise in potential) or hyperpolarization (a drop in potential). The term 1/*Cm*—where *Cm* denotes membrane capacitance—modulates the sensitivity of *V* to current fluctuations: a larger *Cm* slows potential changes for equivalent input currents, mirroring the biological role of membrane capacitance in buffering charge dynamics across the neuronal membrane. On the right-hand side, −*gL*·(*V* − *VL*) acts as the leakage term: *gL* (membrane leakage conductance) governs ion flow through passive membrane channels, while (*V* − *VL*) is the deviation of the current potential from the resting potential (*VL*), driving *V* back to *VL* in the absence of inputs to replicate the “resting state recovery” behavior of biological neurons. *I_exc* (excitatory synaptic current) provides depolarizing drive from upstream activated neurons, while *I_inh_lat* (lateral inhibitory current) and *I_adap* (adaptation current) counteract this drive, with *I_inh_lat* mediating competitive interactions between neighboring neurons and *I_adap* introducing activity-dependent adjustments to the firing threshold. When *V* exceeds the dynamic firing threshold (*Vth*), this equation is temporarily suspended: the neuron generates a spike, *V* resets instantaneously to Vreset (the post-spike resting potential), and remains at *Vreset* during the refractory period (where *dV*/*dt* = 0), simulating the short-term insensitivity of biological neurons post-spiking to prevent overexcitation. The second equation regulates the adaptation current (I_adap), which serves as the primary driver of the neuron’s dynamic threshold by accumulating with spiking activity. This relationship is defined in Equation (2) (this formula is adopted from prior research (Rostami et al. 2022 [32])):

(2) $$\frac{dI_{adap}}{dt}=\frac{1}{\tau_{adap}}\left(-I_{adap}+A_{adap}\cdot spike(t)\right)$$

Here, 1/*tau_adap*—where tau_adap is the adaptation time constant—controls the decay rate of *I_adap*: a larger *tau_adap* prolongs the neuron’s “fatigue state”, matching the gradual fade of adaptation observed in biological neurons (e.g., a *tau_adap* of 100 ms means I_adap takes ~300 ms to decay to near-zero after a spike). The term −*I_adap* ensures *I_adap* fades to zero in the absence of spiking, avoiding persistent elevation of the firing threshold. *A_adap* (the adaptation strength coefficient) determines the magnitude of *I_adap* increase per spike: larger values accelerate threshold elevation during high firing rates, replicating the “neuronal fatigue” phenomenon where frequent spiking makes subsequent activation harder. The *spike*(*t*) function acts as a trigger—taking a value of 1 when a spike occurs at time *t* and 0 otherwise—ensuring *I_adap* accumulates only in response to the neuron’s own activity, aligning with the activity-dependent adaptation of biological neurons. The third equation implements lateral inhibition, a key mechanism for fostering competitive interactions between adjacent neurons to enhance signal contrast in neural networks. Equation (3) quantifies this inhibitory influence and is referenced from Diehl-SNN:

(3) $$I_{inh\backslash \_lat}=w_{lat}\cdot \left(\frac{1}{N_{neigh}}\right)\cdot \sum \left(V_{neigh}-V_{L}\right)$$

In this context, *w_lat* (the lateral inhibition weight) modulates the strength of competitive interactions: larger values intensify inhibition, which is critical for tasks like spatial coding—where strong neighborhood competition ensures only the neuron population most aligned with external inputs remains active. The (1/*N_neigh*) term normalizes the inhibitory effect by the number of neighboring neurons (*N_neigh*), preventing uneven inhibition caused by variations in neighborhood size. The *sum*(*V_neigh* − *VL*) term aggregates the depolarization levels of neighboring neurons: each (*V_neigh* − *VL*) reflects how far a neighbor’s potential deviates from its resting state, meaning more active neighbors (with higher *V_neigh*) generate stronger inhibition on the current neuron. This mirrors the classic “lateral inhibition-enhanced edge perception” observed in the biological visual system, where adjacent cells suppress each other to sharpen sensory representations. These three equations operate in tight coupling: the first integrates inputs and internal states to drive spiking, the second adjusts the firing threshold based on spiking history, and the third mediates inter-neuronal competition. Together, they form a biologically plausible ALIF neuron model that provides a foundational building block for constructing brain-inspired spatial cognition networks.

### 3.2. Designed Lateral Inhibition Distribution Rule

This section focuses on the core innovation: defining the lateral inhibition distribution through two equations to regulate spatial interactions between SOM cluster neuron groups, ensuring adjacent clusters maintain spatial adjacency while avoiding interference from distant clusters, thereby enabling continuous variable encoding. Equation (4) quantifies the total lateral inhibition received by a neuron group at a specific spatial position; it describes the total lateral inhibition *I_inh*(*x*,*t*) that the neuron group at spatial position *x* receives at time *t*. Its calculation relies on an integral over the entire spatial range [−*Ω*, *Ω*], meaning the activity of all other neuron groups in the network contributes to the inhibition of the target group at *x*. Here, *A*(*x′*,*t*) represents the spiking intensity of the neuron group at position *x′* at time t, and *w_inh*(*x*,*x′*) is the lateral inhibition coefficient between the neuron groups at positions *x* and *x′*. The contribution of each neuron group to the target inhibition depends on both its own activity level (*A*(*x′*,*t*)) and the inhibition weight (*w_inh*(*x*,*x′*)) between it and the target group, reflecting the global spatial interaction characteristics of lateral inhibition. This formula is proposed in this work.

(4) $$I_{inh}(x,t)=\int_{-\Omega }^{\Omega }w_{inh}(x,x^{\prime })\cdot A(x^{\prime },t)dx^{\prime }$$

Equation (5) specifies the distance-dependent pattern of inhibition weights, together forming the foundation of the designed lateral-inhibition mechanism. It defines the inhibition weight *w_inh*(*x*,*x′*), which increases monotonically with inter-neuron spatial distance. Parameter *γ* is the upper bound of overall inhibition strength that sets the maximum magnitude of inhibition weight, and ||*x* − *x′*|| denotes the spatial distance between neuron groups at positions *x* and *x′*. The exponential term governs how inhibition weight grows with distance: when *x* and *x′* are close, ||*x* − *x′*|| is small, the exponential term approaches 1 and *w_inh*(*x*,*x′*) stays small, producing weak mutual inhibition for adjacent SOM clusters; when *x* and *x′* are far apart, the exponential term tends toward zero, and *w_inh*(*x*,*x′*) rises toward *γ*, generating strong mutual inhibition among distant clusters. The parameter *σ_inh* controls the growth rate of inhibition: smaller σ_inh accelerates inhibition growth over short separations, while larger *σ_inh* delays it until larger inter-cluster distances. These equations enforce a key spatial rule: adjacent SOM clusters maintain weak inhibition to preserve coordinated co-activation, whereas distant clusters suffer strong inhibition to eliminate undesired cross-talk and guarantee cluster independence. This balance stabilizes cluster-level spatial activation profiles and enables continuous-variable encoding, such as spatial position via smooth activation transitions between neighboring clusters. This formula is proposed herein.

(5) $$\left(w_{inh}(x,x^{\prime })=\gamma \cdot \left[1-exp\left(-\frac{\|x-x^{\prime }{\|}^{2}}{2\sigma_{inh}^{2}}\right)\right]\right)$$

### 3.3. Event-Driven and Temporal Sparse Coding

Event-driven computing and temporal sparse coding form a core paradigm for efficient information processing in brain-inspired systems, offering advantages over traditional clock-driven and rate coding frameworks. Conventional “clock-driven” approaches depend on fixed-time-step sampling, where computations continue regardless of neuronal activity, causing redundant calculations. “Rate coding” conveys information through average firing rates, which needs long-term statistics for stable representation. In contrast, event-driven mechanisms trigger computation only when significant changes happen (like neuronal spiking), while temporal sparse coding focuses information into a few events over time. This combination mirrors the biological nervous system’s on-demand activation and sparse transmission,” cutting down unnecessary computational overhead and boosting information transfer efficiency via precise temporal encoding. To formalize temporal coding, a typical relationship between spike timing and stimulus intensity is expressed as Equation (6). This formula is proposed in this article.

(6) $$t_{spike}=t_{0}-k\cdot \log(I+I_{0})$$

Here, *t_*0 is the start of stimulus presentation; *I* represents the intensity of the input stimulus, and *I_*0 is a threshold current (making sure weak stimuli can still be encoded); and *k* is a time constant that adjusts the sensitivity of spike timing to stimulus intensity. Physically, this relationship embodies the rule that stronger stimuli elicit spikes earlier: High-intensity inputs quickly drive the membrane potential to threshold, while weak inputs need longer integration times. Instead of relying on “rate statistics,” information is encoded via temporal order, keeping fine-grained time resolution and avoiding the need for long-term integration in rate coding.

**Figure 6:**
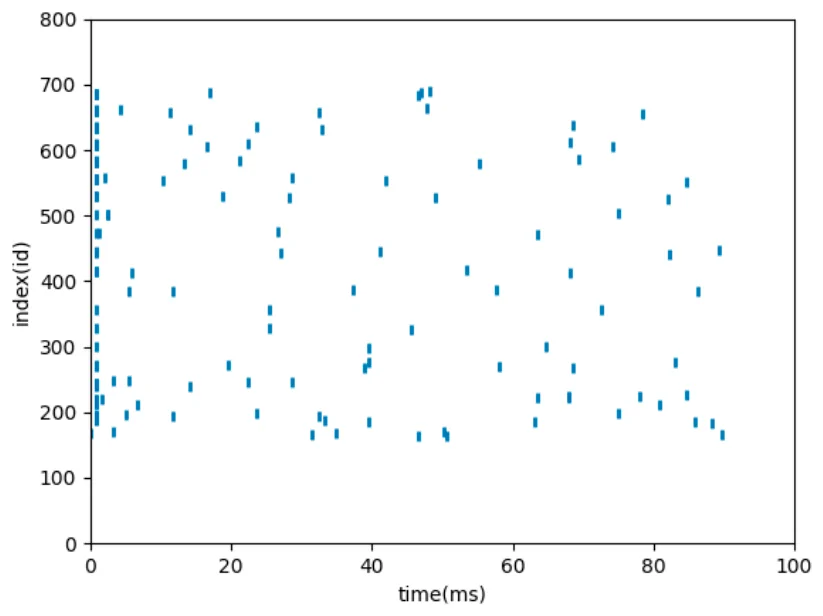
Spatial structural features (contours, textures) of a handwritten digit are encoded as sparse spikes in discrete time windows, via neuron index and spike timing.

**Figure 7:**
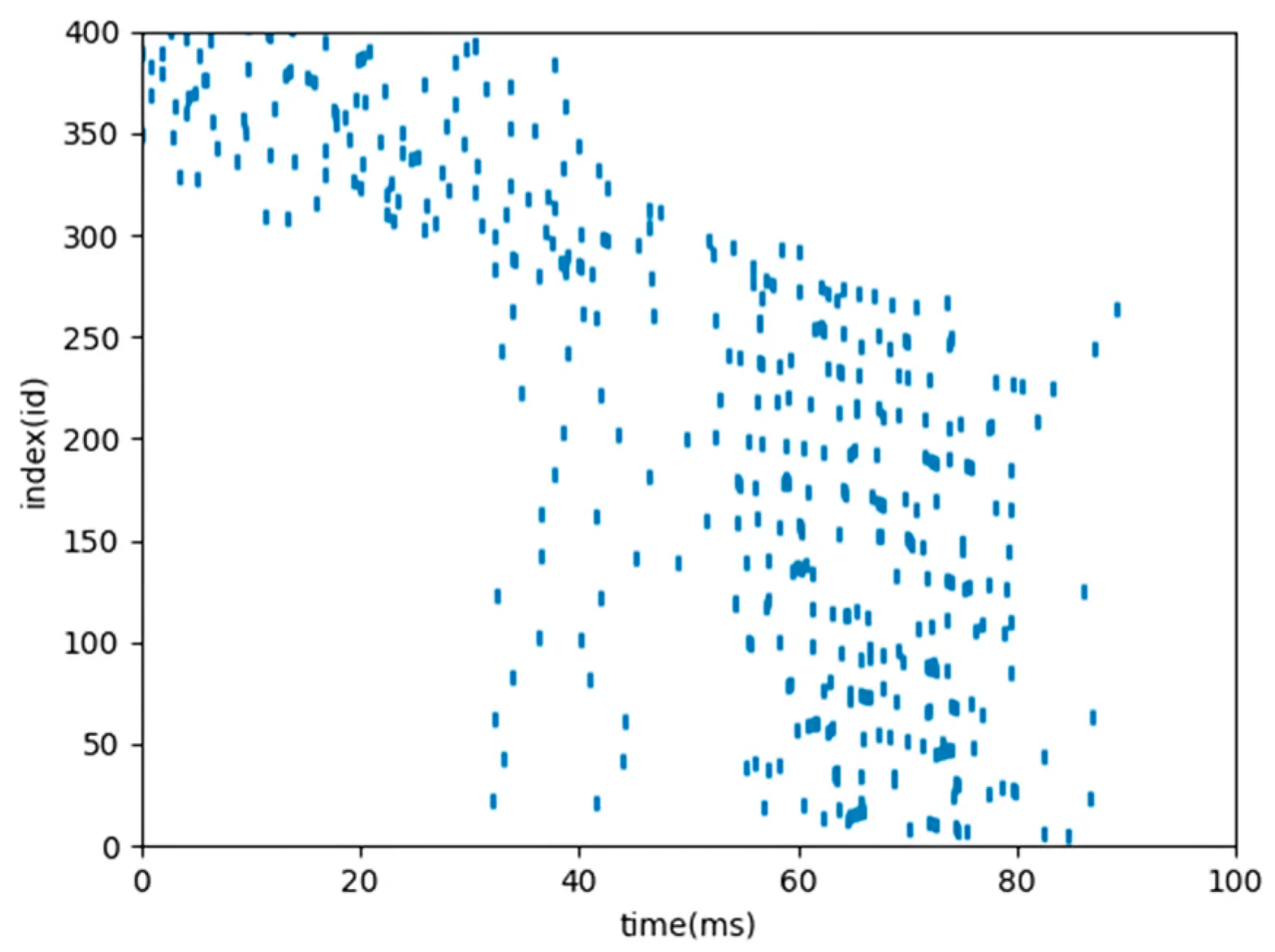
Dynamic spectral components (e.g., consonant-vowel transitions) of a speech sample are encoded as sparse, time-evolving spikes matching speech’s temporal dynamics.

Figure 6 and Figure 7 illustrate temporal coding for MNIST and Spoken-MNIST samples, respectively. Figure 6 (MNIST digit encoding) plots time (ms) on the x-axis and neuron index (corresponding to feature receptive fields) on the y-axis. Neurons encoding digit features do not fire continuously; instead, spikes concentrate in specific time windows (for example, 0–20 ms) and become sparse after that. This temporal sparsity encodes the spatial structure of handwritten digits (like contour and texture positions) via “which neurons spike when”, rather than persistent firing.

Figure 7 (Spoken-MNIST speech encoding) reflects the dynamic nature of speech: While spike distributions are more continuous over time, they remain sparse. Spikes at different time points correspond to distinct spectral components of speech (such as consonant-vowel transitions), and neurons efficiently encode time-frequency features via “timestamp + neuron index”. Compared to clock-driven frame-by-frame computation, event-driven processing only activates when speech features cross neuronal thresholds, greatly reducing computational load and transmission bandwidth.

### 3.4. Neighborhood SOM Clustering Learning Algorithm

Self-Organizing Map (SOM) clustering enables unsupervised competitive learning to project high-dimensional input features onto a low-dimensional topological grid, mimicking the brain’s self-organizing neural representation (Hazan et al. 2025 [33]). A pivotal mechanism is lateral interaction among neurons: when a winning neuron (most responsive to the input) is activated, it inhibits surrounding neurons, with inhibition strength varying with spatial distance—this mirrors biological lateral inhibition and is typically modeled via the Mexican hat function.

(7) $$I_{inh}(d)=A\cdot \exp\left(-\frac{d^{2}}{2\sigma^{2}}\right)-B\cdot \exp\left(-\frac{d^{2}}{2\gamma^{2}}\right)$$

Mathematically, the lateral inhibition strength I_inh as a function of Euclidean distance d from the winning neuron is formulated as Equation (7); this formula is adapted from the Hazan SOM-SNN. In this equation, the first term A·exp(−d^2^/(2·sigma^2^)) captures local excitation (or attenuation of weak inhibition) near the winning neuron (for small d): A scales the strength of excitation, and sigma controls the range where excitation (or weak inhibition) dominates. The second term B·exp(−d^2^/(2·gamma^2^)) represents global inhibition, with B determining inhibition strength and gamma (larger than sigma) governing the range of strong inhibition. Physically, this generates a Mexican-hat profile: neurons very close to the winner experience weak inhibition (or mild excitation), neurons at intermediate distances undergo strong inhibition, and distant neurons see inhibition fade toward a baseline.

**Figure 8:**
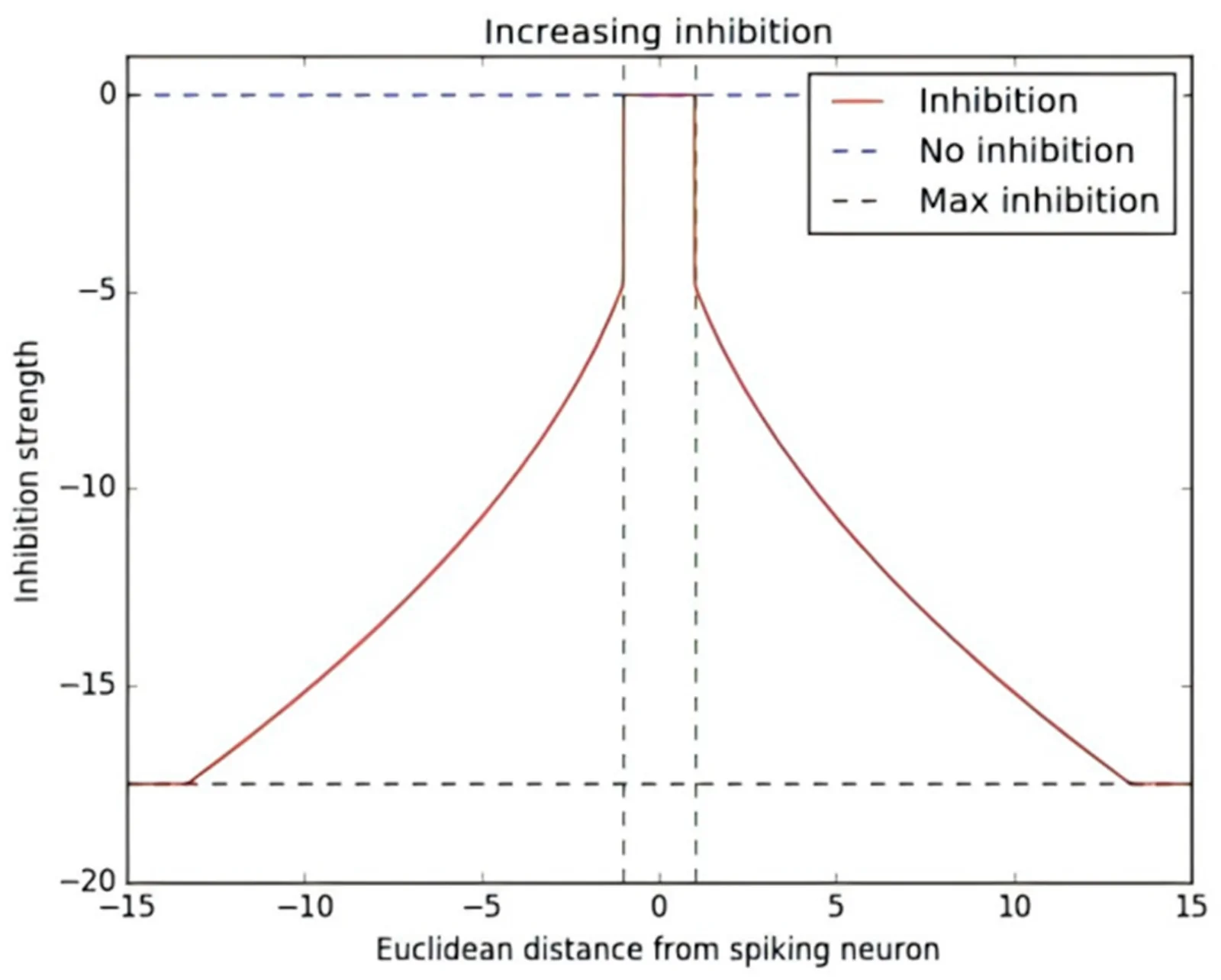
The figure depicts how inhibition strength changes with distance from the winning neuron, generating a Mexican-hat shape with near weak inhibition and distant strong inhibition.

Figure 8 illustrates this relationship: the x-axis denotes Euclidean distance from the spiking (winning) neuron, and the y-axis denotes inhibition strength. The red Inhibition curve shows the net effect: near the winner (distance ~ 0), inhibition is weakest (near the blue dashed No inhibition line); as distance increases, inhibition rises sharply (deeper negative values indicate stronger suppression); and at large distances, inhibition diminishes toward the gray dashed Max inhibition baseline. The green dashed lines mark the effective inhibition zone—within this range, lateral inhibition actively modulates neural competition. This SOM mechanism resonates with CANN’s continuous attractor dynamics: just as CANN sustains a continuous activity bump via distance-dependent recurrent interactions, SOM uses distance-dependent lateral inhibition to enforce topological order—neurons encoding similar inputs cluster spatially, and adjacent neurons compete or inhibit one another. Thus, SOM’s neighborhood clustering not only achieves unsupervised feature organization but also replicates CANN’s core capability to encode and activate continuous neighboring regions, making it a powerful algorithmic analog for brain-inspired representation of continuous variables.

Notably, different from the full-range classical mechanism, the proposed C-SOM SNN adopts the customized inhibition rule in Equation (5) for practical network modulation. This rule corresponds to the initial rising stage of the standard Mexican-hat function, where the lateral inhibition strength consistently increases with Euclidean distance within the effective neighborhood range. In this way, Equation (5) simplifies the complete dual-stage Mexican-hat pattern and retains only the distance-enhanced inhibition characteristic, which realizes progressive competitive suppression among spatially adjacent neurons and fits the topological coding requirement of our spiking network. Equation (7) and Figure 8 intuitively present the full classical Mexican-hat mechanism for comparative illustration, while the actual inhibition rule deployed in our C-SOM SNN follows Equation (5).

### 3.5. Network Structure

**Figure 9:**
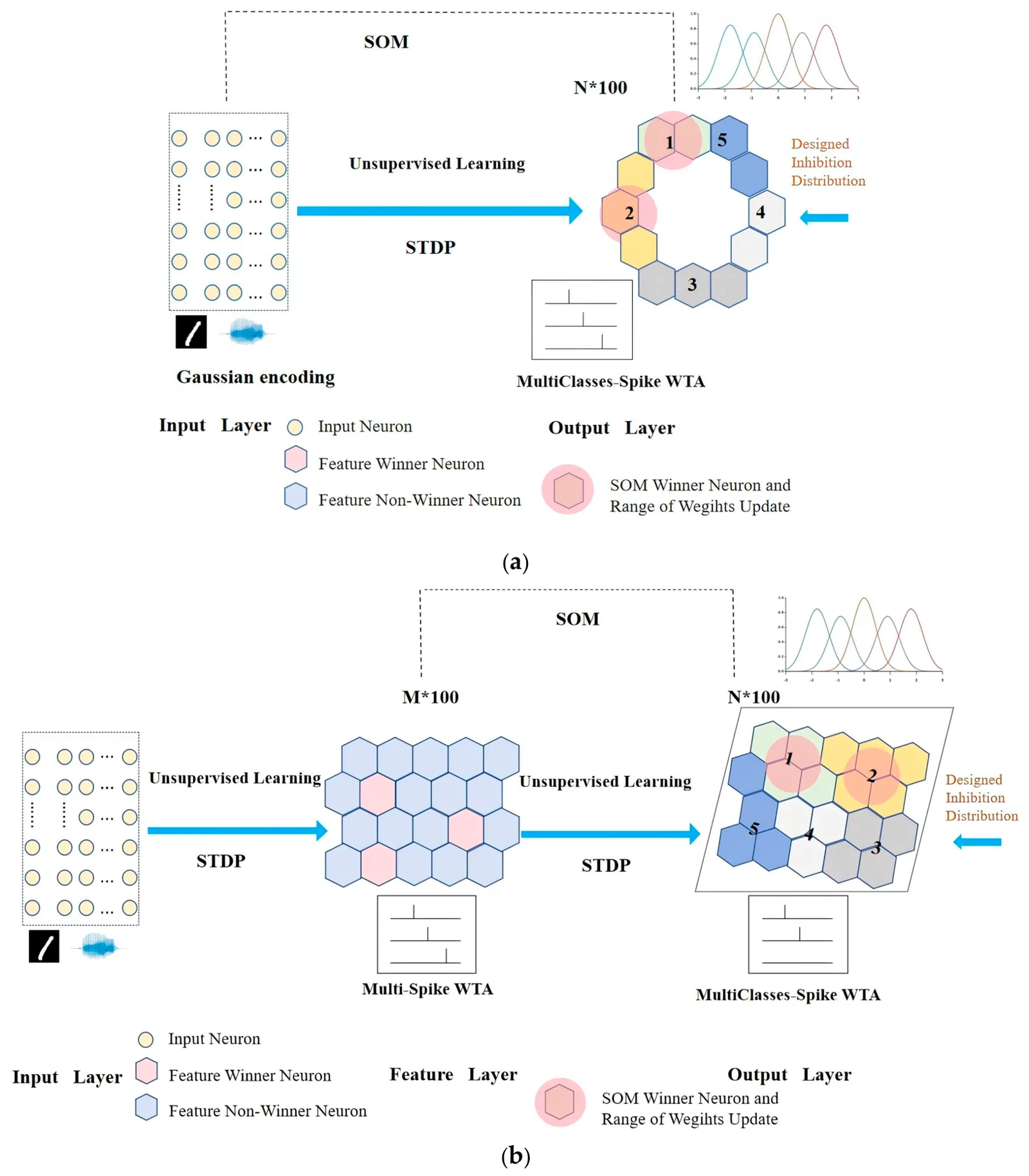
Network structure diagram of 1D ring C-SOM SNN (**a**) and 2D ring C-SOM SNN (**b**). The ring-shaped neuronal arrangement facilitates the effective operation of explicit recurrent connections within output neurons. The differently colored curves in the bump plot represent decoded activity bump profiles under different inputs.

Figure 9a realizes a 1D ring topology continuous attractor with a minimal input–output pair: the input layer converts coordinate data into Gaussian-tuned activity bumps whose width is controllable, directly driving the output layer. Owing to the recurrent ring weights, a stable, shiftable activity bump emerges without any intermediate feature layer, offering both architectural simplicity and canonical continuous-attractor dynamics. It is critical to emphasize that the preset lateral inhibition distribution signal here is achieved through associative learning rather than conventional supervised learning. Specifically, associative synapses project the SOM output spikes from one network to another, enabling cross-modal guidance. In effect, this resembles supervisory feedback; however, it is implemented purely through STDP-based associative learning without labels, gradient descent, or multi-layer backpropagation (Mo et al. 2022 [34]). The activated neuronal population of the source modality naturally guides the target modality’s plasticity through direct spike-timing mapping, analogous to winner-take-all modulation in cortical micro-circuits.

Figure 9b illustrates the hierarchical network architecture integrating SOM and a designed lateral inhibition distribution, consisting of an Input Layer, a Feature Layer, and a specially constructed 2D ring-structured Output Layer. This ring-based topological design is functionally analogous to a 2D continuous attractor neural network. The core design innovations lie in the Output Layer lateral inhibition paradigm, with STDP driving unsupervised learning across all layers. A key feature is the spatially designed lateral inhibition distribution within the Output Layer; its intensity decays with neuron spatial distance (weak for adjacent neurons, strong for distant ones), enabling targeted competition and cooperative activation via recurrent connections. The network employs a Multi-Spike Winner-Take-All (WTA) mechanism: distinct from traditional One-Spike WTA that only allows one neuron to win, 2–11 neurons can form a winning group simultaneously. This Multi-Spike WTA is a relaxed extension of conventional single-neuron One-Spike WTA. Neurons whose membrane potentials exceed the threshold after lateral inhibition and recurrent feedback are selected as winners, and the total number of selected neurons is constrained to 2–11 (Liu et al. 2025 [35]). In the output layer, the adoption of SOM clustering transforms the traditional Multi-Spike WTA mechanism into a Multi-Class-Spike WTA scheme. This enables the sequential activation of two or more neuronal ensembles belonging to distinct categories, thereby realizing a population-level encoding pattern. This feature is of critical importance, as it allows the network to represent complex, category-spanning information through coordinated ensemble dynamics rather than isolated winner neurons. This weakened competition, guided by the designed lateral inhibition, preserves more activation information; winning group neurons encode similar features, balancing clustering distinctness and information retention to enhance clustering precision and robustness. Moreover, the output layer operates in two modes: single-cluster activation yields discrete classification (one bump–one MNIST digit), whereas co-activation of adjacent clusters, permitted by weak local inhibition, forms a continuous activity bump that encodes spatial or temporal variables, unifying discrete clustering and continuous-attractor representation in one substrate.

(8) $$\begin{array}{c} W=\{i|V_{i}(t)+V_{rec}(i)-V_{inh}(i)\geq V_{th}\},2\leq |W|\leq 11 \end{array}$$

(9) $$\begin{array}{cc} \Delta w_{ij} & =I(i\in W)\left[A_{+}exp\left(-\frac{t_{post}-t_{pre}}{\tau_{+}}\right)-A_{-}exp\left(-\frac{t_{pre}-t_{post}}{\tau_{-}}\right)\right] \end{array}$$

Equations (8) and (9) define the coupled unsupervised training algorithm combining Multi-Spike WTA competition and STDP synaptic plasticity. These two formulas are proposed in this work. Equation (8) selects the set *W* of winning output neurons: *i* denotes the neuron index; *V_i*(*t*) is the membrane potential of neuron *i* at time *t*; *V_rec*(*i*) represents recurrent self-feedback potential; *V_inh*(*i*) is the lateral inhibition potential; and *V_th* is the firing threshold. The constraint 2 ≤ |*W*| ≤ 11 limits the number of simultaneously activated winners. Equation (9) computes the synaptic weight update *Δw_ij* from presynaptic neuron *j* to postsynaptic neuron *i*. *I*(*i* ∈ *W*) is the indicator function, enabling STDP only if *i* ∈ *W*. *t_pre* and *t_post* are spike timings of presynaptic and postsynaptic neurons. *A_+*, *A_−* are potentiation and depression amplitudes; and *τ_+*, *τ_*− are the corresponding time constants of the STDP kernel.

### 3.6. Methodology Summary

This subsection provides a structured overview of the methodological components elaborated from Section 3.1, Section 3.2, Section 3.3, Section 3.4 and Section 3.5, concluding the complete technical pipeline of the proposed C-SOM SNN framework. (1) Section 3.1 introduces the adaptive LIF neuron model equipped with lateral inhibition, which governs membrane potential evolution and spike generation for network neurons. (2) Section 3.2 defines the designed lateral inhibition distribution rule, which implements distance-dependent inhibition: weak inhibition for adjacent neurons and strong inhibition for distant neurons to form implicit attractor dynamics. (3) Section 3.3 presents the event-driven and temporal sparse coding scheme, converting raw input signals into spike sequences to fit the temporal computing characteristic of spiking neural networks. (4) Section 3.4 describes the neighborhood SOM clustering learning algorithm, which realizes unsupervised competitive clustering of spike patterns. (5) Section 3.5 specifies the overall ring-arranged network structure, where neurons are placed in a closed ring topology with explicit recurrent connections.

## 4. Results

This section presents some experimental results, including experiments on visual classification and auditory classification with single-group activation, as well as a position encoding experiment in a simulated environment.

Table 1 presents the complete neuronal, plasticity, inhibition, and training hyperparameters of the C-SOM SNN. The neuronal dynamics are configured with a resting potential of −85 mV, a firing threshold of −10 mV, and a refractory period of 5 ms. The asymmetric STDP plasticity is established with a potentiation amplitude of 1.3, a depression amplitude of −0.2, and unified time constants of 20 ms, together with a moderate STDP learning rate of 0.015. The spatial competition is regulated by the lateral inhibition upper bound *γ* of 2.0 and growth rate *σ_inh* of 3.0, ensuring structured topological self-organization during training.

### 4.1. Continuous Variable Encoding of Digits

**Figure 10:**
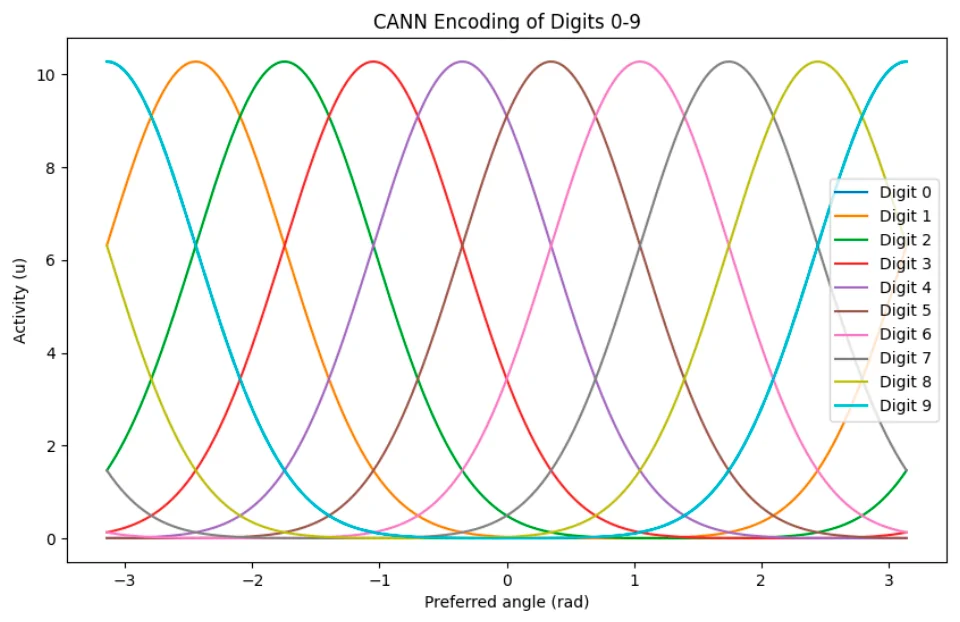
The 1D ring C-SOM SNN encodes the digits 0–9 as a continuum, converting categorical labels into a smoothly shifting one-dimensional activity bump whose spatial phase continuously represents the numerical magnitude.

For continuous data, such as landmark positions or head-direction trajectories, the input stream is inherently repetitive and smoothly varying. Under the C-SOM SNN architecture, this continuity is preserved in the output layer. Figure 10 shows the 0–9 continuum encoded on a 1D ring: activity bumps slide smoothly with preferred angle, so neighboring digits overlap and the spatial activation profile remains continuous, governed by distance-weighted lateral inhibition. Peaks simultaneously supply discrete class labels, yielding a joint discrete–continuous representation within one attractor sheet. Table 2 lists 10 intermediate interpolated inputs and their bump-decoded outputs. This network leverages activity bump positions for continuous representation, resembling Gaussian-based encoding instead of one-hot encoding. The small observed deviations (within 0.05) reflect encoding noise of the ring attractor. The resulting MAE/RMSE of 0.040/0.041 are close to each other, indicating no prominent outlier encoding errors under static stimulus conditions with comparatively little noise.

**Figure 11:**
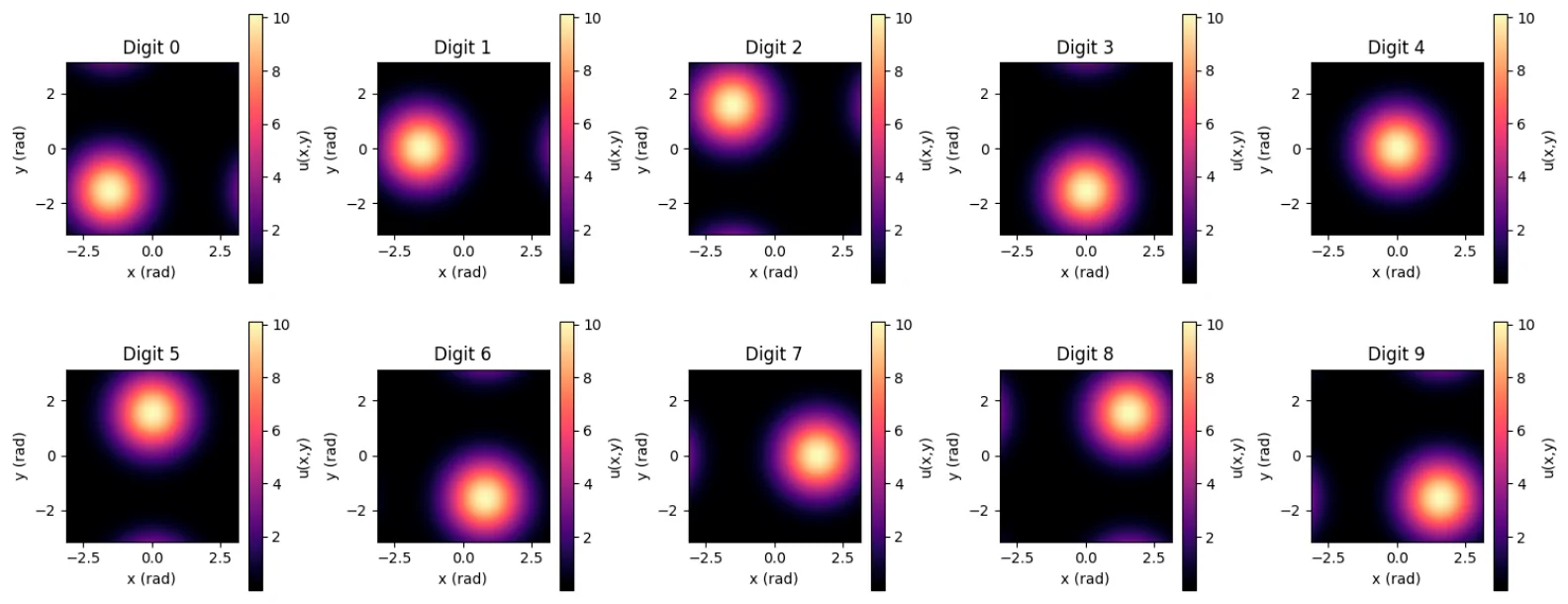
The 2D ring C-SOM SNN encodes the digits 0–9 as a continuum, converting categorical labels into a smoothly shifting two-dimensional activity bump whose spatial phase continuously represents the numerical magnitude.

Figure 11 extends the 1D ring to a 2D ring. Gaussian activity packets translate smoothly in (*x*, *y*) preference space, so neighboring digits remain adjacent, and the bump centroid encodes a continuous class coordinate. Distance-dependent lateral inhibition preserves spatial continuity while peak location still provides an explicit discrete label, yielding a unified 2D continuous-attractor map for digit representation.

### 4.2. Visual Classification Experiment with Single-Group Activation

For discrete pattern-recognition tasks, such as audio or image classification, input samples lack mutual continuity and exhibit large inter-class variability. To preserve the spatially structured lateral-inhibition profile of C-SOM SNN, i.e., to ensure that neuronal ensembles of adjacent categories are activated at neighboring cortical locations, auxiliary spike volleys conveying ground-truth class labels are introduced merely to establish correspondences between neuronal clusters and category memories. These auxiliary label-related spike volleys do not participate in synaptic weight updates during network training (Mo et al. 2026 [36]). Consistent with the unsupervised training algorithm described in Section 3.5, all weight adaptation relies exclusively on the STDP rule gated by multi-spike WTA competition; no backpropagation, gradient computation, or label-driven loss function is adopted to modify synaptic connections. The ground-truth labels are only used after unsupervised self-organization of neural clusters has completed, to assign semantic category identities to the self-formed neuronal groups. The clustering and weight learning process itself remains fully unsupervised and independent of label information. This section presents the visual classification experiment using single-cluster-group activation, targeting handwritten digit recognition with the MNIST dataset. The dataset comprises 60,000 samples for training and 10,000 samples for testing, with classification accuracy evaluated on the test set. Since MNIST images are formatted as 784 pixels, the input layer of the network is configured with 784 neurons, corresponding to individual pixels. Input data is encoded via the temporal coding method introduced in Section 3.3, and the network operates in an event-driven computing mode to ensure efficiency. Additionally, the output layer featuring self-recurrent connections for clustering contains 100 neurons to support single-group activation-based classification.

**Figure 12:**
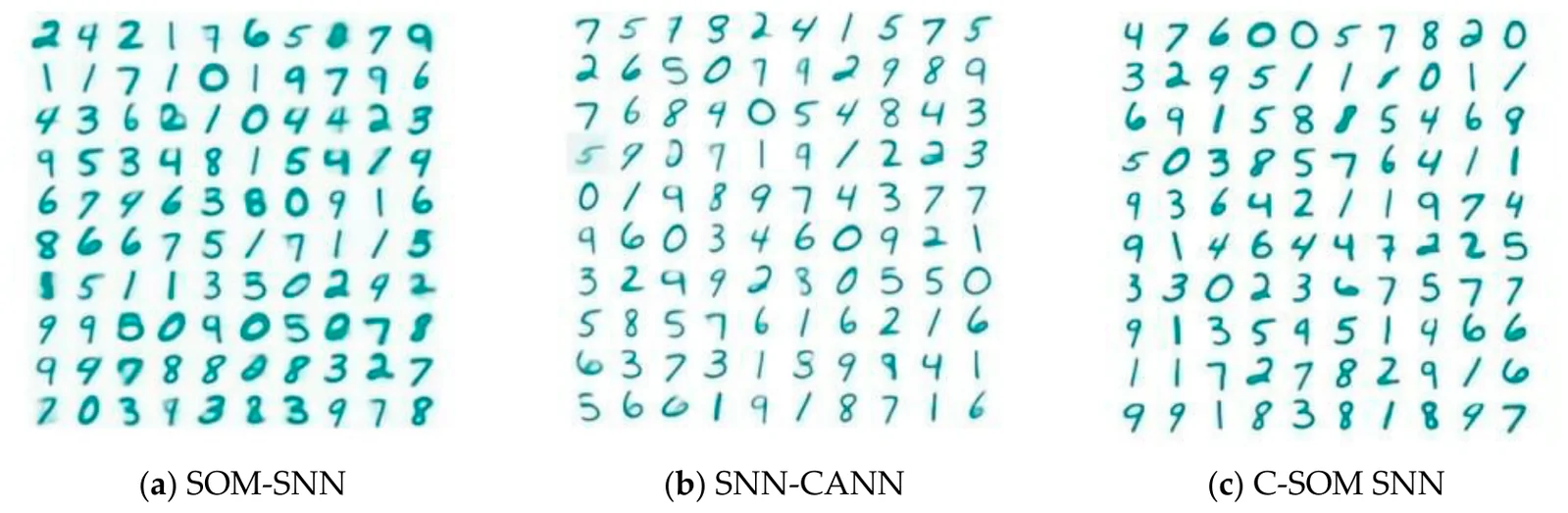
This figure visualizes reconstructed original digits via weight visualization for three networks: classic clustering spiking neural network SOM SNN, SNN-CANN (discussed in Related Work), and the proposed C-SOM SNN.

Figure 12 presents weight visualization results for reconstructing original MNIST digits across three network architectures: the classic clustering spiking neural network SOM SNN, SNN-CANN (discussed in Related Work), and the proposed 2D ring C-SOM SNN. All networks adopt the structural scale of 784-100-100. Baseline models were tuned under the same training protocol to ensure fair comparison. As observed, reconstructed digits from 2D C-SOM SNN exhibit the highest clarity and fidelity: digit contours and textures are sharp and recognizable. In contrast, outputs from SOM SNN and SNN-CANN appear blurrier, with less distinct structural details. This demonstrates that the fusion of SOM-based clustering and CANN-inspired continuous attractor mechanisms in 2D C-SOM SNN enhances the network’s capability to preserve and reconstruct fine-grained visual features, validating the effectiveness of the proposed hybrid framework in visual representation tasks.

**Figure 13:**
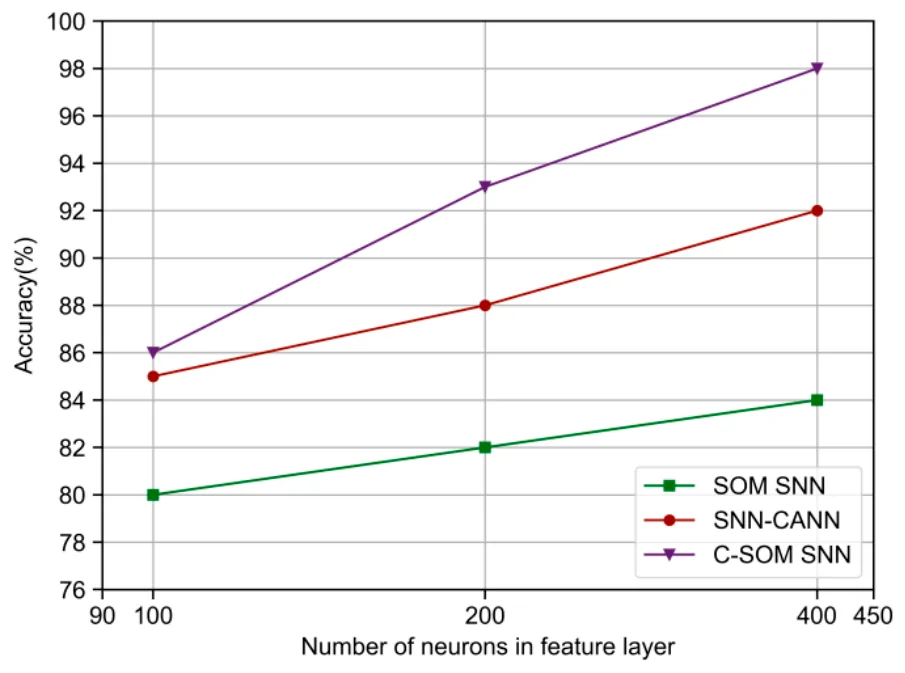
MNIST classification accuracy comparison of SOM SNN, SNN-CANN, and C-SOM SNN with different feature layer neuron numbers.

Figure 13 illustrates the MNIST classification accuracy of SOM SNN, SNN-CANN, and 2D ring C-SOM SNN across three network scales (784-M*100-100, where *M* = 1, 2, 4 for the hidden layer). As the hidden layer neuron count increases, all networks show improved accuracy, yet 2D C-SOM SNN exhibits dominant performance: at 784-100-100, it reaches 86% (vs. 80% for SOM SNN, 85% for SNN-CANN); at 784-200-100, 93% (vs. 82%, 88%); and at 784-400-100, an impressive 98%. Notably, 2D C-SOM SNN achieves near-perfect recognition with only 400 hidden-layer neurons, validating that fusing SOM clustering and CANN-inspired mechanisms endows it with superior efficiency and performance in visual feature representation and classification, surpassing traditional SOM SNN and SNN-CANN significantly.

**Figure 14:**
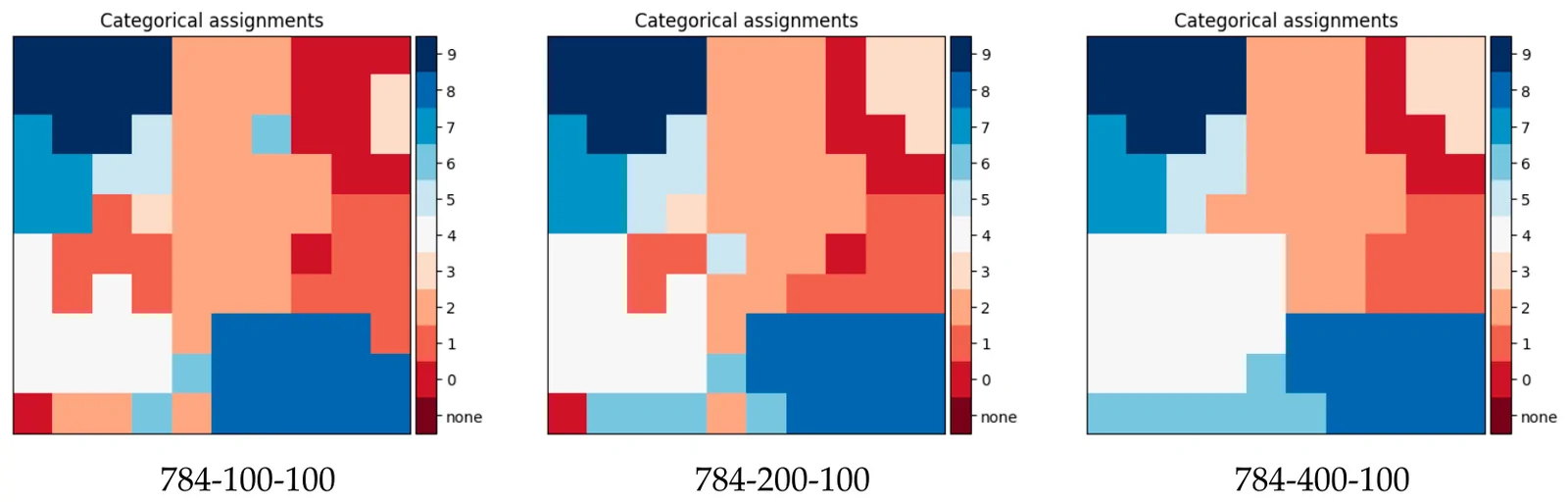
MNIST clustering results of C-SOM SNN under three network scales with different feature-layer neuron numbers.

Figure 14 illustrates the clustering results of 2D ring C-SOM SNN across three network scales (784-100-100, 784-200-100, 784-400-100). Categorical assignments represent different MNIST digit classes, with tighter aggregation of the same color signifying better clustering. As the number of neurons in the feature layer increases, the clustering effect improves markedly: at 784-100-100, class distributions are relatively dispersed; at 784-200-100, same-class regions grow more cohesive; and at 784-400-100, clusters for each digit class become the most compact and distinct. This confirms that increasing the feature layer’s neuron count strengthens 2D C-SOM SNN’s capability to organize and discriminate visual features, thus enhancing clustering performance.

Table 3 lists the classification accuracy (mean ± standard deviation) of SOM-SNN, SNN-CANN and C-SOM SNN under three network architectures. Under the 784-100-100 configuration, SOM-SNN achieves 80.00% ± 0.52% accuracy, SNN-CANN reaches 85.00% ± 0.38%, and C-SOM SNN obtains 86.00% ± 0.30%. When the first hidden layer expands to 200 neurons, the accuracy rises to 82.00% ± 0.45%, 88.00% ± 0.33% and 93.00% ± 0.26%, respectively. Further increasing the first hidden layer to 400 neurons yields accuracies of 84.00% ± 0.40%, 92.00% ± 0.31% and 98.00% ± 0.18%. Relative to the smallest network setting, the accuracy gains of SOM-SNN, SNN-CANN and C-SOM SNN are 4.00%, 5.00% and 12.00%. The results reveal that C-SOM SNN not only maintains the highest accuracy across all architectures but also benefits more from increased hidden-layer capacity. These experiments are repeated five times with a fixed random seed of 42.

**Figure 15:**
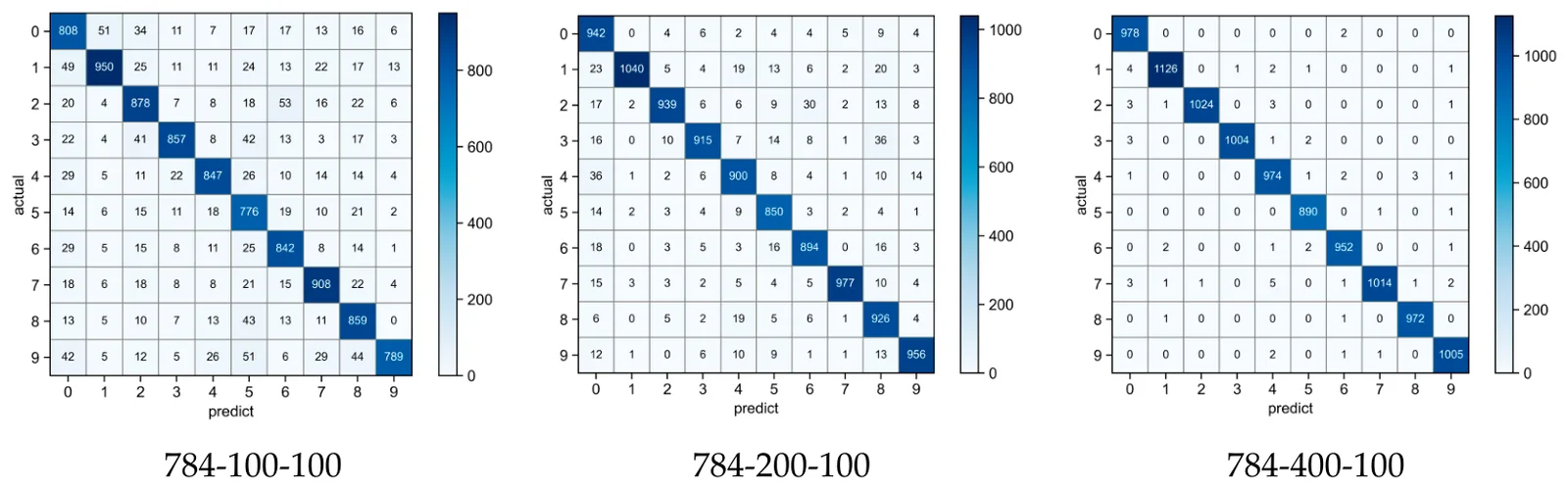
Confusion matrices of C-SOM SNN for MNIST under three network scales with different feature-layer neuron numbers, showing increased correct recognitions with more neurons.

Figure 15 presents the confusion matrices of 2D ring C-SOM SNN under three network scales (784-100-100, 784-200-100, 784-400-100) for MNIST classification, where diagonal entries denote per-class classification accuracy for each digit category. As the number of feature-layer neurons increases, per-class accuracy improves markedly: under the 784-100-100 configuration, the per-class accuracy remains relatively low (e.g., 80.8% for digit 0, 95.0% for digit 1); under the 784-200-100 configuration, per-class accuracy increases substantially (e.g., 94.2% for digit 0); and under the 784-400-100 configuration, most digit classes achieve near-perfect recognition accuracy (e.g., 97.8% for digit 0). This confirms that expanding the feature-layer neuron count enhances 2D C-SOM SNN’s ability to distinguish and recognize digit features, boosting overall classification accuracy.

### 4.3. Audio Classification Experiment with Single-Group Activation

This section focuses on urban sound classification using the Urban8K dataset, which includes 8732 audio samples across 10 categories (e.g., sirens, drills, dog barks) and follows standard training-test splitting protocols. The experimental configuration and hyperparameters are largely consistent with the MNIST visual classification experiment (Section 4.2), such as event-driven computing mode and temporal event coding for input data. To process audio signals, Mel-Frequency Cepstral Coefficients (MFCC) are extracted to capture spectral features. A 700 filter refines features, yielding 100 input-layer neurons matching the filtered dimension, with configuration tailored for MNIST comparability and reduced computational consumption, to feed audio features into the network. Training is conducted with the same epoch count and learning rate as the visual task, ensuring comparability between the two classification scenarios. In addition, given the limited size of the Urban8K dataset, this study follows the official stratified 10-fold protocol on the original 8732 samples. Intra-class Gaussian-noise augmentation is only applied to training subsets after splitting, with test subsets kept unaugmented.

**Figure 16:**
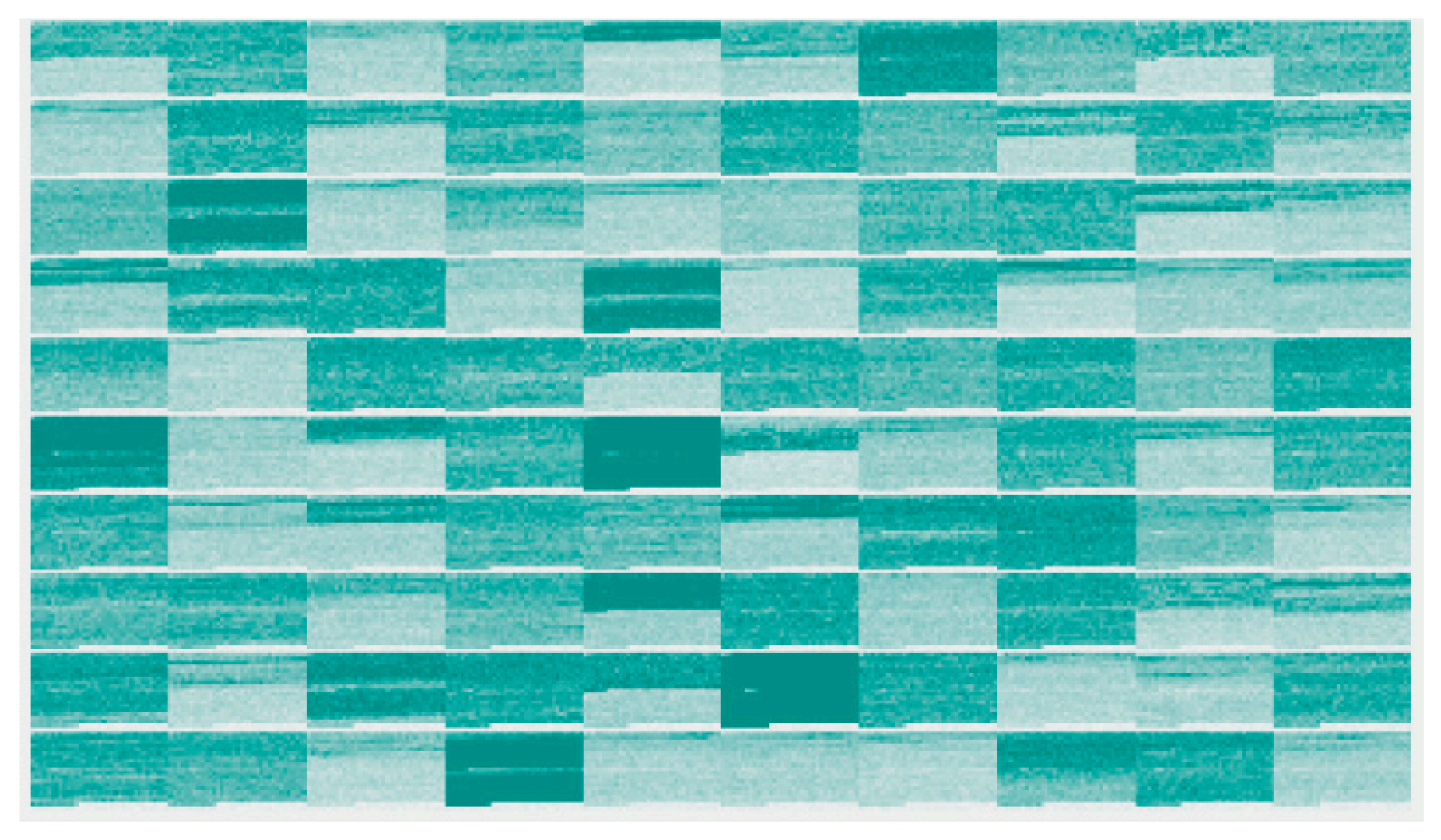
Weight visualization of audio feature spectrograms processed by C-SOM SNN (700-100-100) with audio treated as image information.

As shown in Figure 16, the weight visualization result of audio feature spectrograms processed by 2D ring C-SOM SNN with the structure 700-100-100. In this experiment, audio information extracted via MFCC and refined by filtering (to match the 100-neuron input dimension) is treated as image-like data for network processing. The patterned distribution of shades and blocks reflects the network’s learned representations of spectral features, where variations in color and texture encode distinct auditory attributes (e.g., temporal dynamics of urban sounds). This demonstrates 2D C-SOM SNN’s capability to map audio spectral information onto a visual-feature-like space, integrating auditory feature learning with the network’s clustering and representation mechanisms (originally designed for visual tasks) and laying a foundation for disjoint-cross-modal feature processing in audio classification.

**Figure 17:**
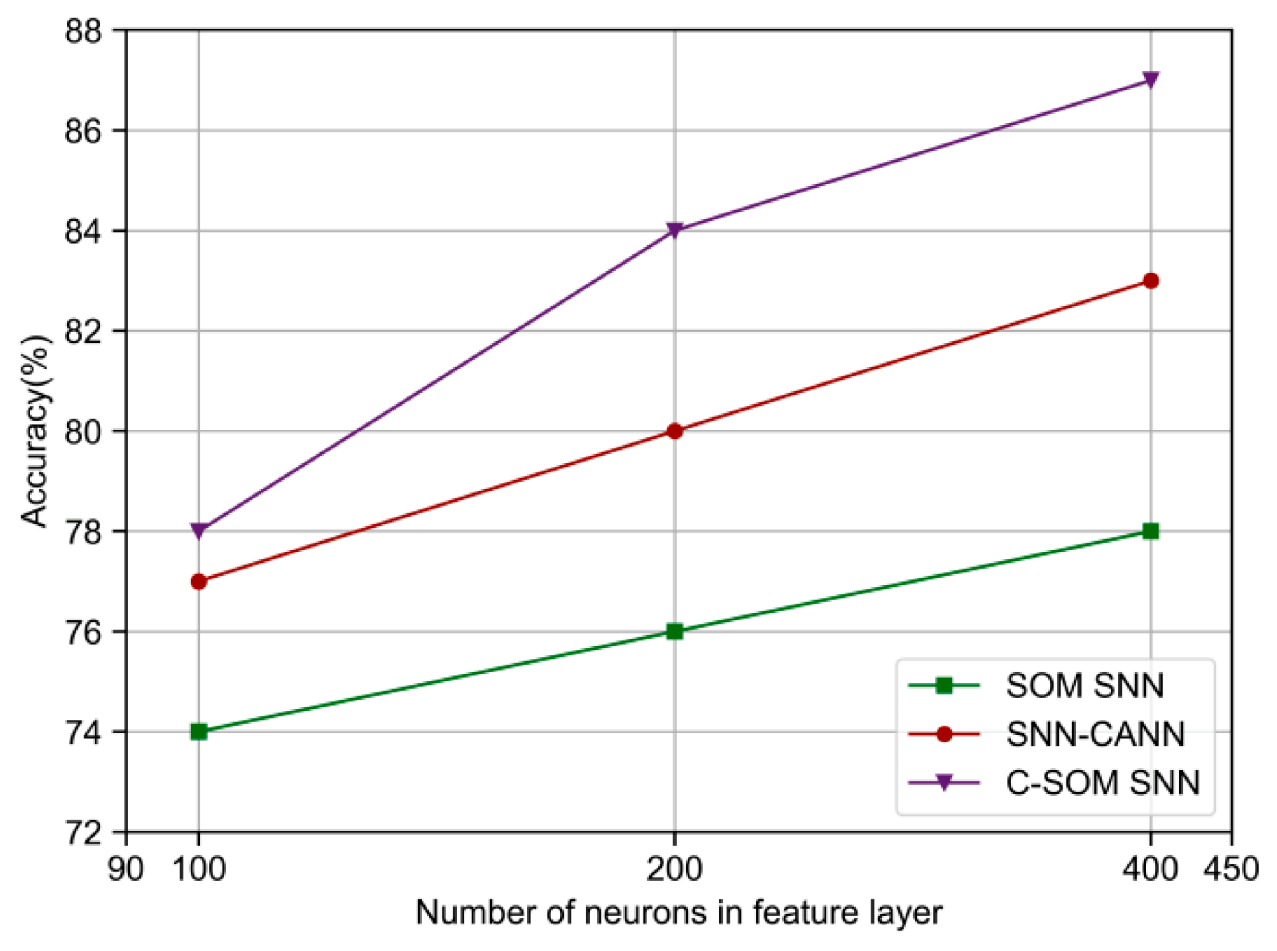
Urban8K audio classification accuracy comparison of SOM SNN, SNN-CANN, and C-SOM SNN with varying feature-layer neuron numbers.

Figure 17 compares the Urban8K audio classification accuracy of SOM SNN, SNN-CANN, and 2D ring C-SOM SNN across three network scales (700-M*100-100, *M* = 1, 2, 4 for the feature layer). As feature-layer neuron counts increase, all models’ accuracy improves, yet 2D C-SOM SNN shows remarkable superiority: at 700-100-100, it reaches 78% (vs. 74% for SOM SNN, 77% for SNN-CANN); at 700-200-100, 84% (vs. 76%, 80%); and at 700-400-100, an impressive 87%. This validates that fusing SOM clustering and CANN-inspired representation empowers 2D C-SOM SNN to better capture audio spectral features, outperforming other SNN variants in audio classification.

**Figure 18:**
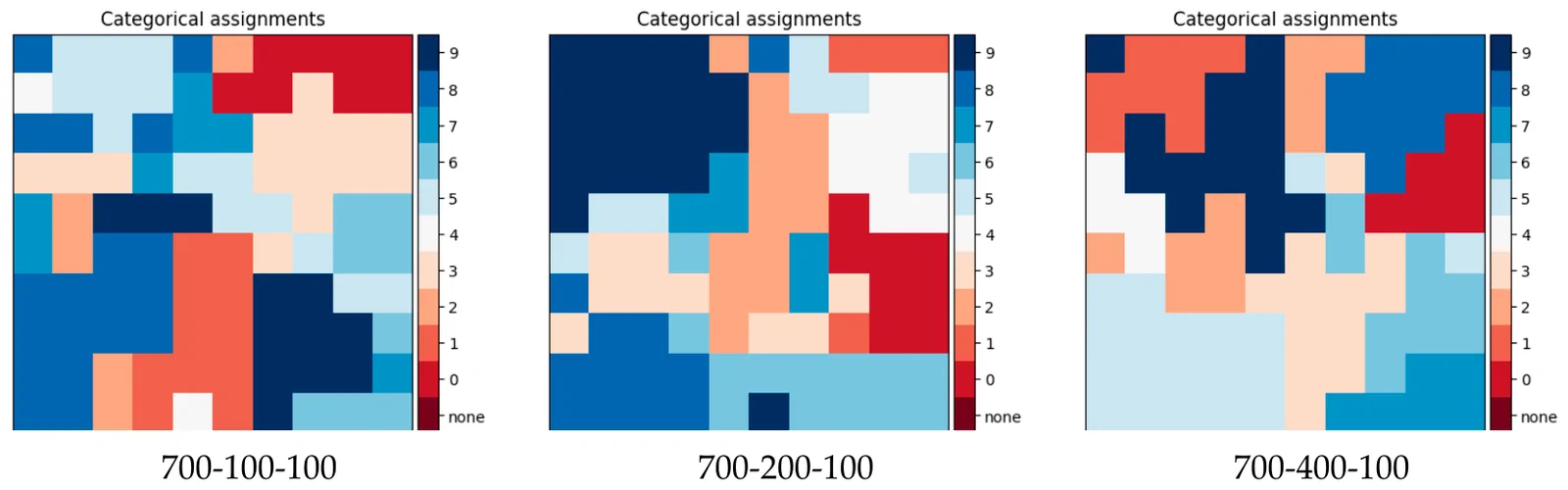
Urban8K clustering results of C-SOM SNN under three network scales with different feature-layer neuron numbers.

As shown in Figure 18, the clustering results of 2D ring C-SOM SNN on the Urban8K dataset across three network scales: 700-100-100, 700-200-100, and 700-400-100. Categorical assignments represent different urban sound classes, with tighter aggregation of the same color indicating better clustering. As the number of feature-layer neurons increases, the clustering effect improves markedly: at 700-100-100, class distributions are relatively dispersed; at 700-200-100, same-class regions grow more cohesive; and at 700-400-100, clusters for each sound class become the most compact and distinct.

Table 4 lists the classification accuracy of SOM-SNN, SNN-CANN and C-SOM SNN on UrbanSound8K under three network architectures. For 700-100-100, the accuracies are 74.00 ± 1.08%, 77.00 ± 0.96% and 78.00 ± 0.82%. When the first hidden layer expands to 200 neurons, the values become 76.00 ± 0.99%, 80.00 ± 0.89% and 84.00 ± 0.75%. At 700-400-100, they reach 78.00 ± 0.92%, 83.00 ± 0.84% and 87.00 ± 0.65%. Compared with the smallest network, C-SOM SNN obtains a 9.00% accuracy gain, larger than the two baselines. These experiments share identical data partitioning and tuning pipelines, with results from 10-fold cross-validation.

**Figure 19:**
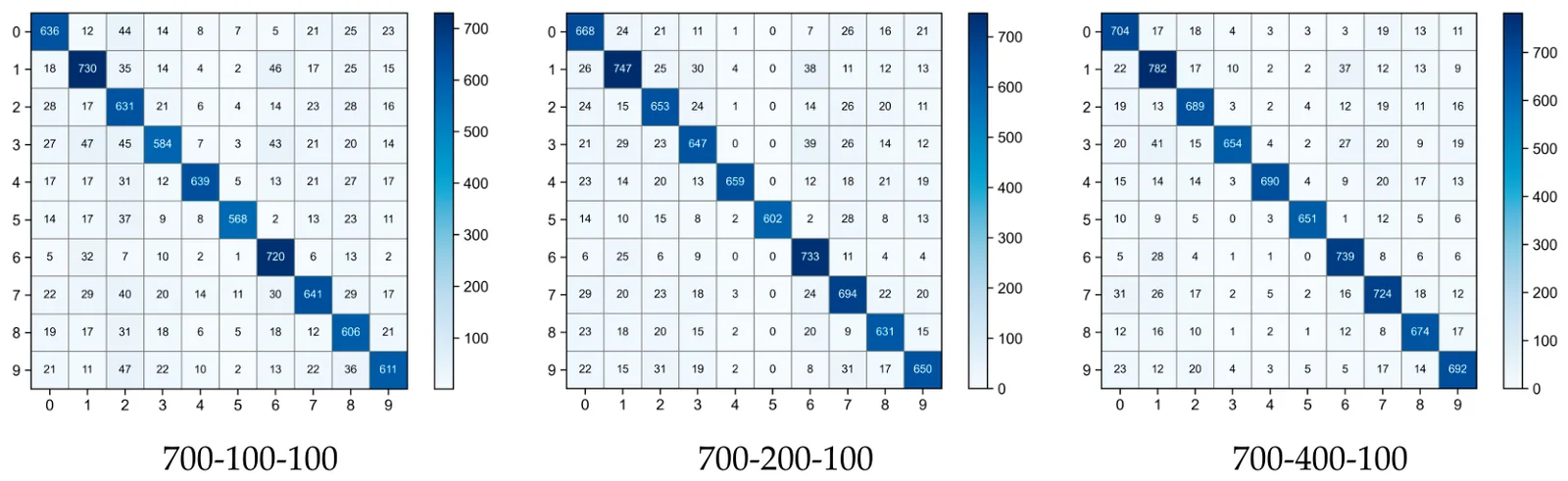
Confusion matrices of C-SOM SNN for Urban8K under three network scales, showing higher recognition rates with more feature-layer neurons.

Figure 19 displays the confusion matrices of 2D ring C-SOM SNN for Urban8K audio classification under three network scales: 700-100-100, 700-200-100, and 700-400-100. Diagonal entries denote the per-class classification accuracy for each audio category. As the number of feature-layer neurons increases, per-class accuracy improves notably: under the 700-100-100 configuration, per-class accuracy remains relatively low (e.g., 63.6% for class 0, 73.0% for class 1); under 700-200-100, per-class accuracy increases (e.g., 68.8% for class 0, 74.7% for class 1); and under 700-400-100, most classes achieve higher recognition accuracy (e.g., 70.4% for class 0, 78.2% for class 1). This confirms that increasing the feature-layer neuron count enhances C-SOM SNN’s ability to distinguish audio features and boosts classification performance.

C-SOM SNN integrates the self-organizing clustering capability of SOM with a designed lateral inhibition distribution, adopting the biologically plausible unsupervised STDP learning rule for model training. This integration breaks the limitation of traditional SNN variants that mostly focus on discrete variable encoding.

**Figure 20:**
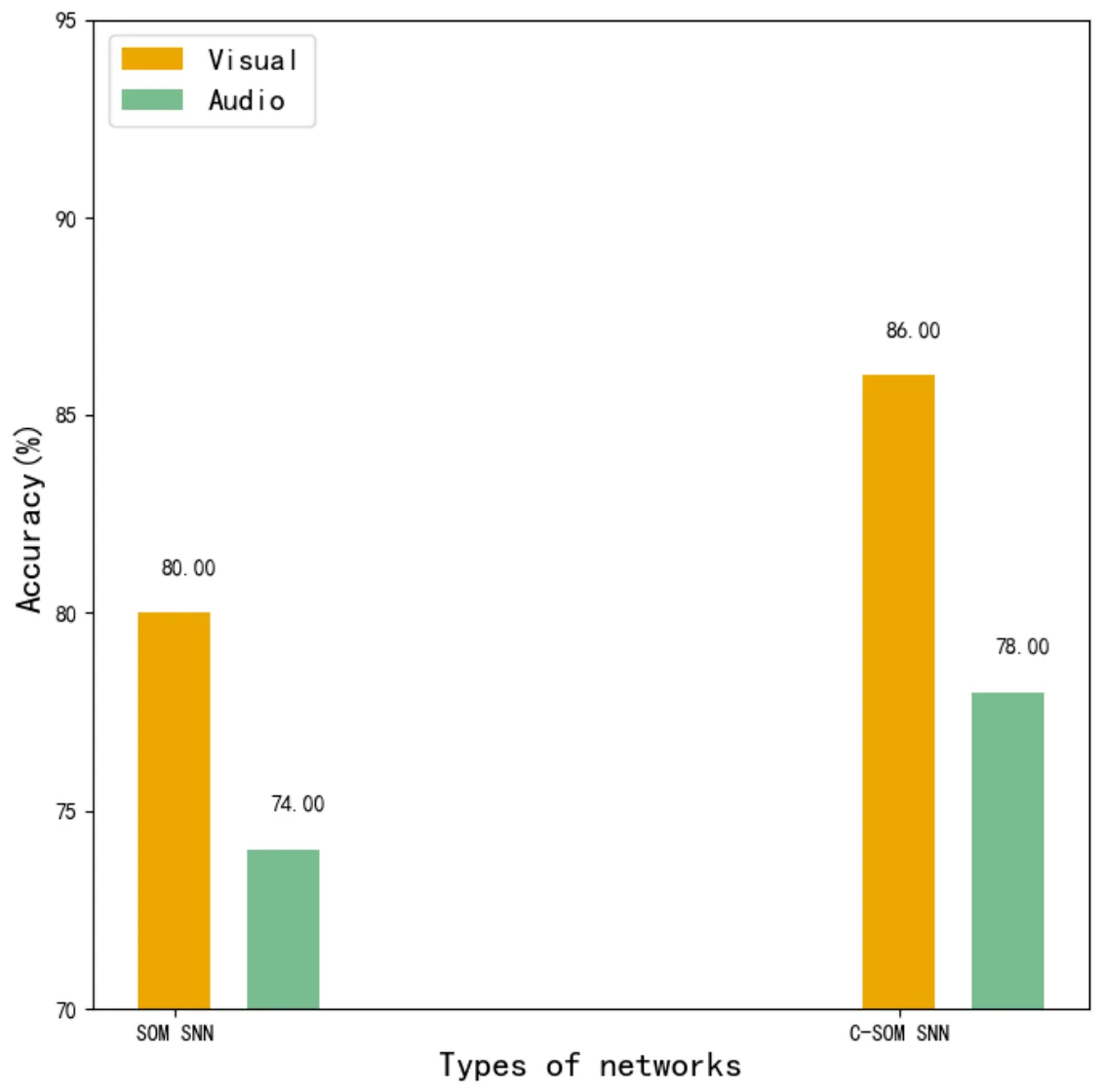
Classification accuracy comparison of SOM SNN, C-SOM SNN on MNIST(visual) and Urban8K (audio) datasets.

By leveraging the customized spatial decay of lateral inhibition intensity (weak inhibition for adjacent neurons, strong inhibition for distant ones), C-SOM SNN enables effective representation for both discrete and continuous variables within a single architecture using a learnable weight set. When lateral inhibition is strong, the network operates as a discrete encoder that assigns sparse, orthogonal spike codes to discrete categories; when inhibition is tuned to moderate levels, adjacent clusters are co-activated, integrating local activations into a continuous manifold for smooth encoding of spatially continuous variables. This dual-mode encoding flexibility eliminates the traditional trade-off where models are restricted to either discrete classification or continuous mapping. As shown in Figure 20, under the network configuration of 784-100-100, 2D ring C-SOM SNN significantly outperforms SOM SNN on both visual (MNIST dataset) and audio (Urban8K dataset) tasks. On visual classification, C-SOM SNN achieves 86.00% accuracy compared to 80.00% for SOM SNN; on audio classification, it reaches 78.00% versus 74.00% for SOM SNN. This consistent improvement across modalities validates that fusing SOM clustering and the designed lateral inhibition mechanism endows the network with superior capability to preserve and reconstruct fine-grained features. The hybrid topology combining SOM competitive dynamics with distance-dependent lateral inhibition ensures that similar inputs trigger synchronous spiking in adjacent neuron clusters while distant regions are suppressed, yielding output spike clusters that are spatially continuous, limited in quantity, and stable in position, a combination that achieves topological preservation and noise robustness rarely seen in existing spiking network models.

### 4.4. Ablation and Sensitivity Analysis for Classification Tasks

Table 5 quantifies the individual and combined contributions of the designed lateral inhibition and Multi-Spike WTA modules. Removing either component degrades classification accuracy on both datasets. Using standard fixed inhibition reduces MNIST (784-400-100) accuracy by 4.80% and Urban8K (700-400-100) accuracy by 3.90%. Switching to single-neuron WTA leads to a smaller performance drop. When both modules are disabled, the model achieves the lowest accuracy. The results verify that the two proposed modules jointly boost classification performance.

Table 6 analyzes the parameter sensitivity of the 784-400-100 C-SOM SNN. The model achieves its peak accuracy of 98.0% at the default *γ* and *σ_inh*. A small *γ* reduces accuracy to 95.6% due to insufficient inhibition, while a large *γ* suppresses neurons and degrades performance to 96.1%. A small *σ_inh* drops accuracy to 97.2%, and a large σ_inh compresses coding space, leading to 96.7% accuracy.

### 4.5. Time Consumption and Robustness Analysis for Classification Tasks

Table 7 compares the computational efficiency of our event-driven C-SOM SNN with the clock-driven SNN from Diehl-SNN on the MNIST dataset, measured on an NVIDIA RTX 4090 (24 GB GPU). Our event-driven model achieves much lower time consumption across all network sizes. For 784-100-100, the training time per epoch is 5 min versus 17 min; for 784-200-100, 7 min versus 33 min; for 784-400-100, 11 min versus 61 min. This efficiency gain arises from spike-triggered computation and sparse spike activity, which avoids redundant calculations for silent neurons. The advantage tends to grow as the first hidden layer expands, indicating favorable scalability of the event-driven design. These results demonstrate improved computational efficiency under the tested setup, suggesting potential suitability for low-power neuromorphic deployment. Future energy savings may be achieved on dedicated neuromorphic chips or FPGAs.

Table 8 compares the noise robustness of SOM-SNN, SNN-CANN and the proposed C-SOM SNN with the 784-400-100 network architecture. Gaussian white noise of specified intensity is injected only into test-set samples. Under clean test inputs, C-SOM SNN reaches 98.00 ± 0.18%, outperforming SNN-CANN (92.00 ± 0.31%) and SOM-SNN (84.00 ± 0.40%). As noise intensity increases, all models suffer accuracy decay. At noise = 0.3, C-SOM SNN still maintains 91.2 ± 0.5%, with the smallest performance drop and variance, verifying its superior robustness against noisy visual inputs.

### 4.6. Multi-Group Activation Localization and Navigation Experiment

This experiment aims to leverage 1D ring C-SOM SNN for encoding and representing continuous landmark positions, focusing on simulating the location-aware capability of a machine agent in a navigable space. The input data of the network is the (*x*, *y*) coordinate information of landmarks: the maximum value of the horizontal coordinate *x* is 251, and the maximum value of the vertical coordinate *y* is 131, so the number of input-layer neurons is set to 382 (matching the total dimension of coordinate information). When the machine agent stays at a specific position, 2–3 landmarks are visible in its field of view; it conducts continuous learning three times at the same position, with each learning session lasting 2 time units. When the agent moves, it advances one grid step each time, and due to the continuity of the movement path, it can still see the landmarks observed at the previous position—this results in continuous overlapping of the network’s input data. The feature layer is configured with 300 neurons to extract position-related feature information from the input coordinate data. The output layer, also with 300 neurons, generates group pulse firing patterns to characterize different landmarks. Since the landmarks are distributed continuously in the space, the network thereby achieves the encoding and representation of continuous position variables, laying a foundation for subsequent localization and navigation tasks.

**Figure 21:**
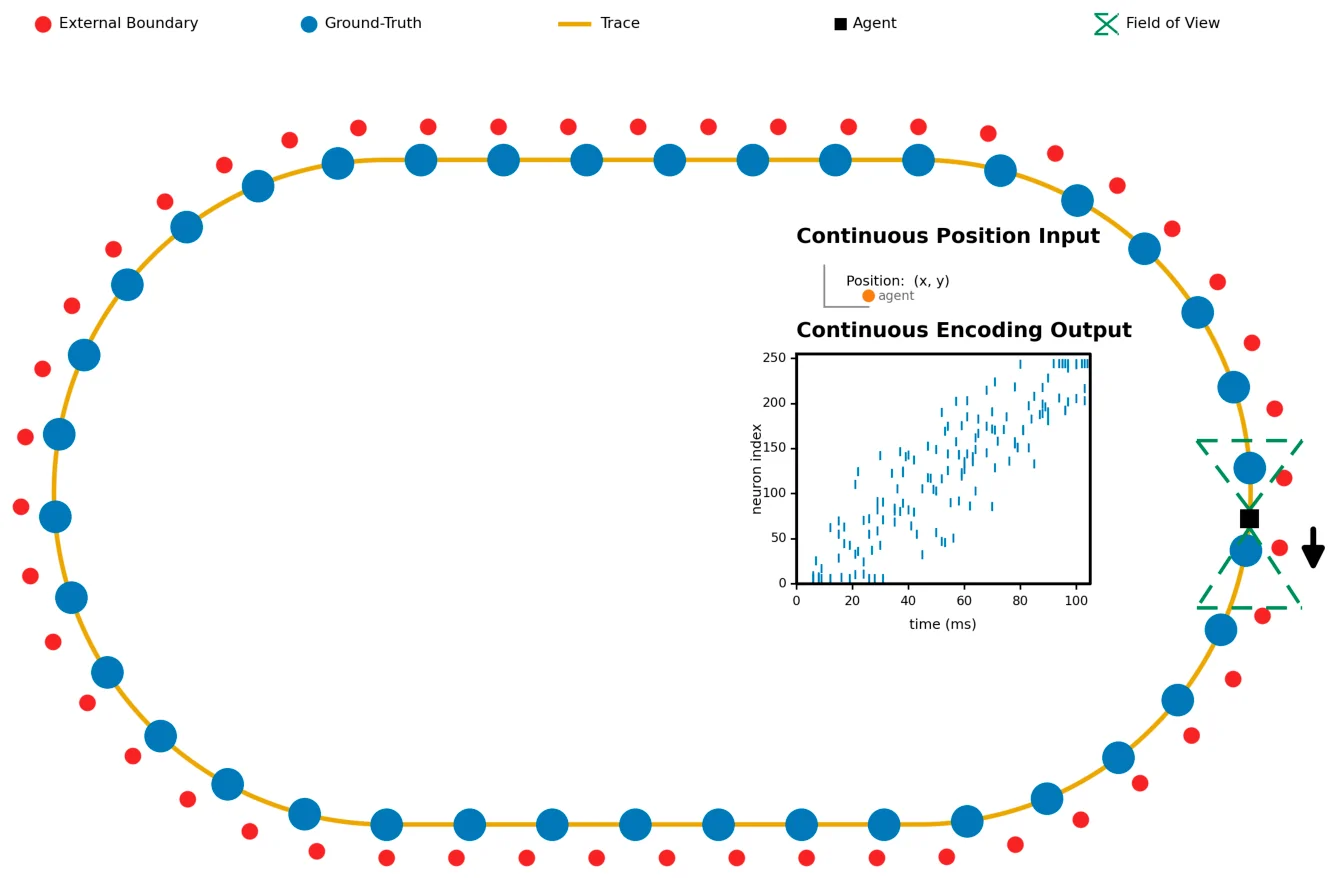
C-SOM SNN-based localization and navigation simulation with continuous position input, continuous encoding output, and spike train representation.

Figure 21 presents a 1D ring C-SOM SNN localization and navigation simulation. Red dots mark the external boundary, and blue dots represent the ground-truth track. The yellow trace completely overlaps with the ground-truth track, demonstrating that the predicted trajectory precisely matches the actual path. The black square indicates the agent, with a green dashed diamond showing its field of view. Above the track, the schematic illustrates the experimental pipeline: continuous position input (*x*, *y*) is fed into the network, which performs continuous encoding output via population spike trains, enabling the agent to stably encode and navigate along the continuous path through repeated sensory input. This experiment demonstrates that C-SOM SNN possesses significant potential for localization and navigation tasks.

**Figure 22:**
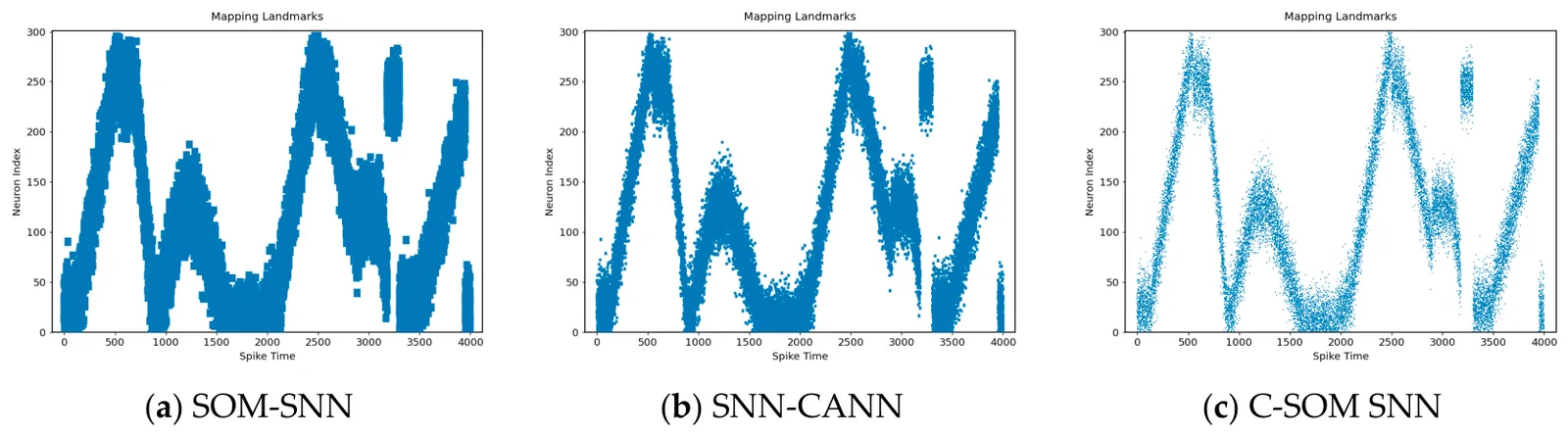
Comparison of output-layer landmark representation capability among SOM SNN, SNN-CANN, and C-SOM SNN.

Figure 22 compares the output-layer capability of SOM SNN, SNN-CANN, and 1D ring C-SOM SNN in representing landmark information. At the same spike time, adjacent neurons activate—driven by continuously repeated inputs, along with the output layer’s clustering/steady-state operations and self-connections of output neurons that bolster stability. Moreover, thinner lines signify more stable neurons and better convergence: C-SOM SNN exhibits the thinnest lines (optimal stability and convergence), SNN-CANN is intermediate, and SOM SNN has the thickest lines (least stable), verifying C-SOM SNN’s superiority in encoding continuous spatial landmarks.

Table 9 compares localization performance of SOM-SNN, SNN-CANN and the proposed 1D ring C-SOM SNN on training trajectories and unseen novel trajectories. MAE and RMSE measure the mean and root-mean-square coordinate errors, where higher values indicate worse localization. The 1D C-SOM SNN achieves train MAE/RMSE of 1.02/1.31 and novel MAE/RMSE of 1.47/1.86, consistently lower than SOM-SNN and SNN-CANN. All models yield larger errors on unseen trajectories owing to distribution shift. The significant error reduction confirms that our 1D C-SOM SNN learns robust spatial representations for continuous localization and navigation.

## 5. Discussion

The performance gains observed in C-SOM SNN can be physically interpreted by the distance-dependent lateral inhibition mechanism. Weak inhibition between neighboring neurons preserves local spike correlation, while strong long-range inhibition suppresses redundant spike activities. This spatial inhibition profile naturally shapes implicit attractor dynamics, enabling simultaneous encoding of discrete categories and continuous spatial variables without hand-crafted recurrent attractor connections. When benchmarked against SOM-SNN, Diehl-SNN and SNN-CANN, the proposed model achieves superior classification accuracy on MNIST and Urban8K and delivers smoother continuous position encoding than SNN-CANN. SOM-SNN supports spike clustering but lacks structured lateral inhibition for continuous representation. Diehl-SNN relies heavily on static STDP rules optimized only for discrete classification and lacks support for continuous variable representation, whereas SNN-CANN requires predefined recurrent weights to maintain attractor states and suffers from higher computational overhead. Several limitations of this work should be acknowledged. All experiments are conducted via software simulation, and the network has not been deployed on neuromorphic hardware. Visual and audio tasks are processed as separate disjoint modalities, with no cross-modal feature fusion implemented. Continuous landmark encoding tests are performed on simulated navigation datasets rather than real-world robot trajectories. Model behaviors are also sensitive to selected hyperparameters, including *γ* and *σ_inh*. Variations of these parameters alter the decay rate of lateral inhibition and may degrade representation stability when values deviate from the validated range. From the perspective of brain-inspired navigation, the distance-dependent inhibition provides a simplified computational analogy of the inhibitory microcircuits within the medial entorhinal cortex, revealing how structured local inhibition can support stable population coding of spatial locations. Nevertheless, substantial gaps remain between the simulated results and real robotic deployment. Future work will address these constraints by hardware implementation on neuromorphic chips, exploring true multi-modal fusion mechanisms and validating continuous spatial encoding under physical robot navigation scenarios.

## 6. Conclusions and Future Work

In this work, a novel spiking neural network architecture termed C-SOM SNN was developed. Integrating the self-organizing clustering capability of SOM SNN with customized lateral inhibition distribution, the model was trained via biologically plausible unsupervised STDP learning rules. This framework overcame the inherent limitations of traditional SNN variants that only support discrete variable encoding. Distinct from conventional CANN attractor models that rely on predefined global recurrent weights to form stable attractor states, the proposed C-SOM SNN adopts a purely ring-structured topology for the output layer. Specifically, the output neurons are equipped with explicit self-recurrent connections and configurable lateral inhibition distributions, establishing topological coding rules without relying on fixed attractor weight configurations. With spatially decaying lateral inhibition that imposes weak inhibition on adjacent neurons and strong inhibition on distant neurons, effective representation of both discrete and continuous variables was realized. Systematic experimental validations verified the effectiveness of the proposed model. On the MNIST visual dataset and Urban8K audio dataset for discrete category classification tasks, higher classification accuracy was yielded by the C-SOM SNN compared with SOM SNN and other conventional unsupervised SNNs. In the localization navigation task for continuous landmark encoding, stable representation of continuous spatial positions was established via group spike firing modulated by the designed lateral inhibition, which effectively eliminated the coding discontinuity inherent in discrete representation schemes. The introduction of STDP learning further improved the biological interpretability of the model, which conformed to the developmental trend of brain-inspired intelligence. Nevertheless, model evaluations in this study were conducted based on relatively single-modality and medium-scale datasets. For future research, the model framework will be extended to larger-scale disjoint-multi-modal datasets, such as audio-visual paired datasets, to comprehensively validate its generalization performance. In addition, disjoint-multi-modal synergy mechanisms will be further explored. Subsequent investigations will focus on the optimization of lateral inhibition distributions for disjoint-cross-modal feature integration through clustering and associative learning, aiming to simulate the efficient multi-sensory information processing characteristics of biological brains.

## Figures and Tables

**Table 1:** Corresponding training parameters of the C-SOM SNN.

Parameter	Value
Neurons’ resting potential	−85 mV
Neurons’ threshold	−10 mV
Neurons’ decay rate	0.9
Neurons’ refractory period	5 ms
STDP potentiation amplitude	1.3
STDP depression amplitude	−0.2
STDP potentiation time constant	20 ms
STDP depression time constant	20 ms
STDP Learning rate	0.015
Lateral inhibition upper bound	2.0
Lateral inhibition growth rate	3.0
Time steps	100 ms
Epochs	4
Batch size	64

**Table 2:** Continuous representation for intermediate interpolated values.

Ground-Truth	Bump-Decoded	Ground-Truth	Bump-Decoded
0.6	0.64	5.4	5.37
1.2	1.24	6.3	6.26
2.7	2.66	7.8	7.75
3.5	3.53	8.5	8.46
4.1	4.15	9.2	9.16
MAE/RMSE	0.040/0.041		

**Table 3:** Classification accuracy MNIST with different network architectures.

Scale	SOM-SNN (%)	SNN-CANN (%)	C-SOM SNN (%)
784-100-100	80.00 ± 0.52	85.00 ± 0.38	86.00 ± 0.30
784-200-100	82.00 ± 0.45	88.00 ± 0.33	93.00 ± 0.26
784-400-100	84.00 ± 0.40	92.00 ± 0.31	98.00 ± 0.18

**Table 4:** Classification accuracy Urban8K with different network architectures.

Scale	SOM-SNN (%)	SNN-CANN (%)	C-SOM SNN (%)
700-100-100	74.00 ± 1.08	77.00 ± 0.96	78.00 ± 0.82
700-200-100	76.00 ± 0.99	80.00 ± 0.89	84.00 ± 0.75
700-400-100	78.00 ± 0.92	83.00 ± 0.84	87.00 ± 0.65

**Table 5:** Ablation comparison of C-SOM SNN network on the MNIST and Urban8K dataset.

Designed Inhibition	Multi-Spike WTA	MNIST (%)	Urban8K (%)
Yes	Yes	98.00 ± 0.18	87.00 ± 0.65
No	Yes	93.20 ± 0.35	83.10 ± 0.81
Yes	No	94.60 ± 0.29	84.40 ± 0.76
No	No	88.70 ± 0.48	79.30 ± 0.94

**Table 6:** Lateral-inhibition parameter sensitivity analysis for C-SOM SNN on the MNIST.

*γ* (*σ_inh* = 3.0)	MNIST (%)	*σ_inh* (*γ* = 2.0)	MNIST (%)
0.5	95.6	1.0	97.2
1.0	96.8	2.0	97.8
2.0 (default)	98.0	3.0 (default)	98.0
3.0	97.4	4.0	97.5
4.0	96.1	5.0	96.7

**Table 7:** Comparison of time consumption for training 1 epoch on the MNIST dataset.

Model	Network Size	Driven Method	Time Consumption (min)
Diehl-SNN	784-100-100	Clock-Driven	17
	784-200-100	Clock-Driven	33
	784-400-100	Clock-Driven	61
C-SOM SNN	784-100-100	Event-Driven	5
	784-200-100	Event-Driven	7
	784-400-100	Event-Driven	11

**Table 8:** Classification accuracy under different input noise levels on the MNIST dataset.

Model	Clean (%)	Noise = 0.1 (%)	Noise = 0.3 (%)
SOM-SNN	84.00 ± 0.40	80.2 ± 0.6	73.5 ± 0.9
SNN-CANN	92.00 ± 0.31	88.5 ± 0.5	81.7 ± 0.8
C-SOM SNN	98.00 ± 0.18	95.7 ± 0.3	91.2 ± 0.5

**Table 9:** Localization error comparison across different network architectures.

Model	Train MAE	Train RMSE	Novel MAE	Novel RMSE
SOM-SNN	3.42	4.15	5.88	7.02
SNN-CANN	2.35	2.98	3.91	4.76
C-SOM SNN	1.02	1.31	1.47	1.86

## Data Availability

The original contributions presented in this study are included in the article, and further inquiries can be directed to the corresponding author.

## Abbreviations

The following abbreviations are used in this manuscript:

SNN	Spiking Neural Network
STDP	Spike-Timing-Dependent Plasticity
SOM	Self-Organizing Map
CANN	Continuous Attractor Neural Network
WTA	Winner-Take-All
LIF	Leaky Integrate-and-Fire
MFCC	Mel-Frequency Cepstral Coefficients
SLAM	Simultaneous Localization and Mapping
AGV	Automated Guided Vehicle
